# The enteric DNA virome differs in infants at risk for atopic disease

**DOI:** 10.1080/19490976.2026.2616066

**Published:** 2026-01-27

**Authors:** Tyrus J. Perdue, Cassandra E. Newkirk, Robert Beblavy, Antti E. Seppo, Erin C. Davis, Michael B. Sohn, Kirsi M. Järvinen, Cynthia L. Monaco

**Affiliations:** a Department of Microbiology and Immunology, University of Rochester Medical Center, Rochester, NY, United States; b Department of Medicine, Division of Infectious Diseases, University of Rochester Medical Center, Rochester, NY, United States; c Department of Biostatistics and Computational Biology, University of Rochester Medical Center, Rochester, NY, United States; d Division of Allergy and Immunology, Center for Food Allergy, Department of Pediatrics, University of Rochester School of Medicine and Dentistry, Golisano Children’s Hospital, Rochester, NY, United States; e Department of Medicine, Division of Allergy, Immunology, and Rheumatology, University of Rochester School of Medicine and Dentistry, Rochester, NY, United States

**Keywords:** Atopic disease, bacteriophage, infants, enteric virome, phageome

## Abstract

Atopic disease prevalence, including atopic dermatitis, food allergy, asthma, and allergic rhinitis, has risen dramatically in industrialized countries. Traditional, single family farming lifestyles protect against atopic disease, but the mechanisms are incompletely understood. While there are established epidemiologic connections between childhood respiratory viral infections and the infant gut bacterial microbiome with allergic disease development, the influence of the early enteric virome on atopic disease development is unknown. We analyzed the enteric virome in 131 infants from high-atopy-risk urban/suburban environments and low-atopic-risk single-family farming communities. While similar at 12 months, enteric bacteriophage communities significantly differed by farm-life versus urban lifestyle at six weeks and six months of age. A lifestyle protective from atopic disease demonstrated higher colonization rates of *Bifidobacterium longum* subsp. *infantis* (*B. infantis*), an important beneficial commensal, with phageome communities differing in infants colonized by *B. infantis* at all time points. Simultaneously, *Mastadenovirus* and *Bocaparvovirus* were more prevalent in urban infant stools at six months of age. Sparser phage-phage networks were found at all timepoints in infants who later developed atopic disease. These data suggest that the early infant enteric DNA virome develops differently in farming and urban lifestyles and may factor into risk of atopic disease development.

## Introduction

Atopic diseases, including atopic dermatitis (AD), food allergy (FA), asthma, and allergic rhinitis, are chronic inflammatory conditions characterized by an exaggerated IgE-mediated response to a foreign antigen.^1-3^ These typically develop in early infancy and childhood and can persist lifelong.^4^ AD and FA are often the first to manifest, with around 8% of the US pediatric and 4% of the adult population suffering from FA, the leading cause of anaphylaxis.^5^ AD and FA are both independent risk factors for the later development of other atopic diseases in the so-called “atopic march.”^6^ While heredity is a strong determinant of atopic disease, the rapid increase in disease prevalence in recent decades suggests underlying environmental, dietary, and microbial exposures associated with a Western lifestyle influence disease onset.^7-10^ Prior studies examining Amish, Old Order Mennonite (OOM) and Hutterites have shown that a traditional, single-family farming lifestyle is associated with protection against development of atopic diseases.^5^^,^^11-19^ Individual factors identified as protective against atopic diseases include consumption of unpasteurized farm milk,^11-13^ exposure to farm animals and stables, and a larger number of siblings.^5^^,^^20^ Repeated, consistent exposure to diverse microbes during infancy and early childhood may train the immune system to a tolerogenic response upon later exposure to food antigens and thereby modulate risk of FA and other atopic diseases.^19^ An increasing body of evidence shows that the gut bacterial microbiome (bacteriome) plays an important role in the health of children, providing nutrients and metabolites necessary for barrier function and development of a healthy immune system.^21^^,^^22^ Alteration from a healthy gut bacteriome is associated with development of atopic diseases including AD, FA, and asthma.^5^^,^^19^^,^^23-25^

There is an established epidemiologic link between development of atopic disease and early childhood viral infections,^19^ likely through alterations of the developing host immune response and/or mucosal barrier function. The virome, or viral microbiome, is comprised of two main groups of viruses: bacteriophages and eukaryotic viruses.^26^ Both are able to elicit an immune response that can impact localized inflammation as well as trigger adaptive immunity.^27^^,^^28^ Perturbations of the virome composition have been associated with pediatric diseases such as diarrhea and malnutrition, as well as inflammatory bowel disease and type 1 diabetes.^19^^,^^25^ Bacteriophages comprise the majority of the virome, infect and replicate inside bacteria and thus play important roles in shaping bacterial communities by selective predation or horizontal gene transfer, which can confer new functions to infected bacteria.^29-34^ Abnormal response to viral infection in early infancy could be one factor leading toward the development of atopic disease.^35^ Early respiratory eukaryotic viral infections including those caused by RSV and rhinovirus are associated with onset of allergic rhinitis and asthma during childhood.^35-39^ Atopic individuals exhibit deficient interferon antiviral response in multiple immune cell lineages to a variety of viral infections and stimuli,^35^ although the initiating event – whether viral infection or immune dysfunction from atopy – is unknown. Studies on the impact of daycare on atopic risk suggest that very young infants may be more susceptible to eukaryotic viral infections with prolonged hyperresponsiveness.^40^^,^^41^ Bacteriophages may also contribute to the development of atopic disease.^42-45^ A recent study examining gut DNA bacteriophages in 1-y-old children showed differences in bacteriophage populations in those who later developed asthma, independent of bacterial populations.^45^ However, the role of the infant enteric virome in early development of atopic disease has not been previously studied.

Herein, we compare enteric DNA viruses over time from 131 infants in two disparate cohorts: OOM single-family farming communities with a low rate of atopic diseases^17^ and Rochester, New York infants from urban/suburban areas (ROC) with a first-degree relative with atopic disease and therefore at a higher risk of developing atopic disease.^46^ A prior cross-sectional study of this community found that not only were the infants from the farming lifestyle at a lower risk for atopic disease, but also the fecal bacteriome composition differed between the groups.^18^ We hypothesized that infants born to communities with different lifestyles and at different risk for atopic diseases would differ in their specific bacteriophage community composition. We show that lifestyles with disparate risk for atopic disease development show enteric DNA bacteriophage and eukaryotic virus populations that diverge in early infancy, demonstrating an association between the enteric virome composition and atopic disease risk.

## Materials and methods

### Study population

**Figure 1.**
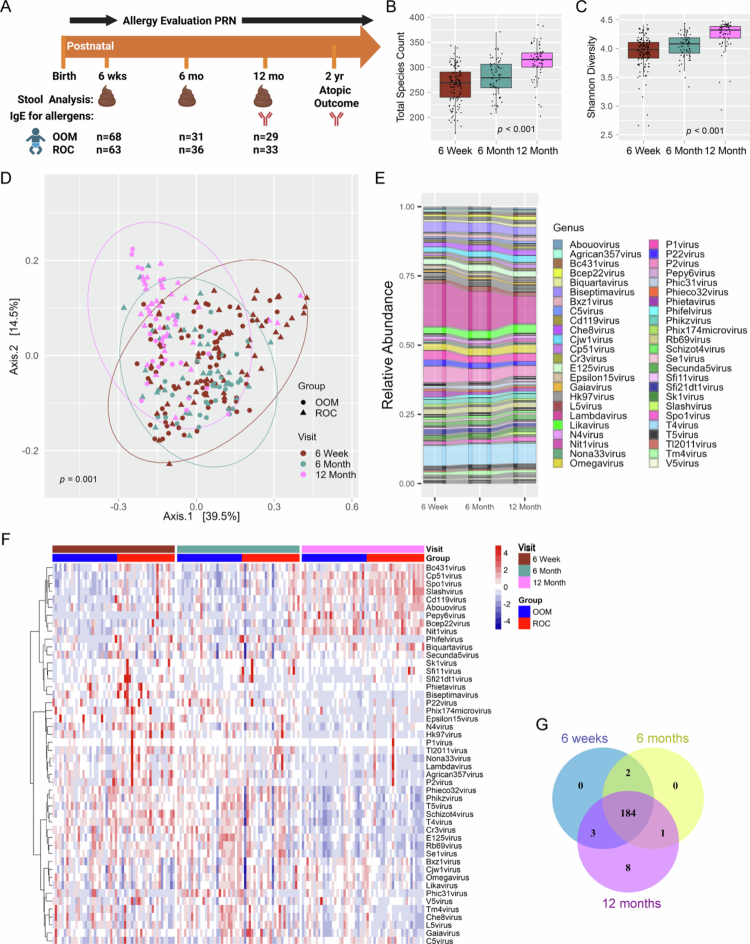
The enteric phageome changes during the first year of life. (A) Timeline of sample collection, analysis, and outcome for ZOOM cohort. Adapted from.^46^ Figure made in BioRender. (B) Total bacteriophage species count and (C) bacteriophage genus-level Shannon diversity of all subjects at each visit. (D) Principal coordinate analysis of bacteriophage genera in all samples across the three visits as measured by the Bray‒Curtis dissimilarity index. Circles = OOM, triangles = ROC. (E) Alluvial diagram of bacteriophage genus relative abundance across all samples (6 weeks *n* = 131, 6 months *n* = 67, 12 months *n* = 62) over time. The top 48 most abundant bacteriophage genera over visits are colored; the remaining are gray. (F) Heatmap of the 40 most abundant bacteriophage genera at each visit (48 total genera) from the 53 infants that had virome data available at all three timepoints, arranged by visit and cohort. Order of infants is the same at each timepoint. The top bar indicates lifestyle group, the bottom bar indicates collection time. Relative abundances were normalized to unique reads and centered and scaled for improved visualization, zero representing mean relative abundance. Ward's hierarchical clustering was utilized (y axis). *n* = 53 per visit; OOM, *n* = 28; ROC, *n* = 25. (G) Venn diagram of shared core taxa between timepoints, defined as those genera present in at least 20% of samples and with a relative abundance ≥ 0.0001.

Pregnant women were recruited prenatally during the second and third trimesters from the OOM of Western New York and urban/suburban Rochester, NY (ROC) populations as part of the “Zooming in on Old Order Mennonites” (ZOOM) cohort as previously described.^46^ The OOM are from Penn Yan, New York, and immediate surroundings, roughly 65 miles southeast from Rochester, New York, as described before.^46^ Pregnant OOM women were recruited via their midwife/birth attendant and through word of mouth, and the pregnant Rochester (ROC) women were recruited from allergy practices found within the Rochester, NY area and from pediatric and OB-GYN practices as well as over social media. In-person visits were performed on all infants at 6 weeks, 6 months, 12 months, 18 months, and 24 months of age, as described before.^46^ These ROC infants all had a first-degree relative with a diagnosed or self-reported atopic disease. Methods, demographics, and initial atopic outcomes for this cohort have been published.^46^ For this study, we utilized samples and data from 68 OOM and 63 ROC infants at the six-week timepoint and from a smaller subset of infants with and without atopic outcomes at subsequent timepoints (Figure 1A). Two ROC infants included in the six-week analyzes had no 24-month follow-up, so their atopic outcomes at twelve months (nonatopic) were used.

### Sample collection

Stool samples were collected at each study visit. Mothers were provided with instructions, a stool collection kit, and diaper liners for the infants. Once infant stool was passed, the sample was transferred to a sterile tube using a sterile, disposable spatula and immediately frozen in the home freezer and transported frozen to the laboratory by courier or study coordinator where it was then stored at −80 °C.

### Assessment for infant outcomes of allergic diseases

The first 1000 d of life appears to be a critical window for FA development; therefore, we have focused on this period.^5^ Details regarding assessment of infant outcomes of allergic diseases are detailed in Järvinen et al., 2022.^46^ In brief, allergy symptoms were assessed via surveys and during in-person study visits performed on all infants at 6 weeks, 6 months, 12 months, 18 months, and 24 months of age by the investigator and/or subinvestigators. Any children in both cohorts who displayed symptoms of allergic disease, such as chronic rash, hives, food reactions, food intolerance, vomiting, diarrhea, bloody stools, wheeze/persistent cough, and chronic rhinitis, were evaluated by the study physician, and allergen-specific IgE tests (ImmunoCAP, ThermoFisher), skin prick tests and oral food challenges, as appropriate to refute or confirm food allergy, were performed guided by these symptoms. The diagnosis of atopic dermatitis (AD) was based on recurring or chronic red, pruritic rash in a distribution age-typical for AD. Food allergy (FA), including non-IgE-mediated FA, was diagnosed based on the NIAID sponsored expert panel guidelines on diagnosis and management of FA.^47^ In addition, all children had blood drawn for specific IgE to major food and aeroallergens at 12 and 24 months of age. Food or aeroallergen sensitization was defined by a positive skin prick test or specific IgE. Children who had either FA and/or AD were considered to have atopic disease.

### DNA extraction and shotgun metagenomic sequencing

Only infants that completed their 12-month visit and had an available stool sample for analysis were included in this study, culminating in 68 OOM and 63 ROC infants. DNA extraction and sequencing were performed by Diversigen, Inc. (Minneapolis, MN). DNA was extracted from roughly 250 µg of infant stool using the DNeasy PowerSoil Pro Kit (Qiagen) automated for high throughput on a QiaCube HT (Qiagen), with bead beating in Qiagen Powerbead Pro plates (Cat No. 19311). DNA was then quantified with the Qiant-iT Picogreen dsDNA Assay (Invitrogen). Libraries were prepared using the Nextera Library Prep kit (Illumina) with a protocol adapted from manufacturer's instructions. Libraries were then sequenced on an Illumina NextSeq using paired-end 2 × 150 reads with a NextSeq 500/550 High Output v2 kit (Illumina) at a sequencing depth of 20 million paired-end reads. Average total paired end reads per sample were 23,347,909.

### Virome taxonomic identification

DNA metagenomic sequencing data were input into the VirusSeeker virome pipeline,^48^ which is a validated bioinformatic pipeline for viral taxonomic identification.^49^^,^^50^ Briefly, raw sequences underwent preprocessing, including adapter removal, read stitching, quality filtering including removal of low-complexity sequences, and CD-HIT, to remove redundant sequences (98% identity over 98% of the sequence length). Sequences then underwent human genome filtering. Unmapped sequences were sequentially queried against a custom virus database comprised of all viral sequences in NCBI (download date August 3, 2020) using BLASTn (e-value cutoff 1 × 10^−10^), followed by BLASTx (e-value cutoff 1 × 10^−3^), yielding a pool of “candidate viral sequences.” False-positive viral sequences were removed from this pool by successively querying candidate viral sequences using MegaBLAST (e-value cutoff 1 × 10^−10^) against the NCBI NT database (download date August 3, 2020), with unidentified sequences then queried using BLASTn against the NCBI NT database, and any remaining unidentified sequences queried using BLASTx (e-value cutoff 1 × 10^−3^) against the NCBI NR database (download date August 30, 2020). Sequences that aligned to viruses in the above queries at the designated e-value threshold were taxonomically classified as bacteriophage or eukaryotic virus based on the NCBI identity of the top hit.^48^ The average total viral reads assigned per sample was 67,078 (0.287% of total paired-end reads). Average percentage reads assigned to eukaryotic viruses compared to total quality control reads were 0.019%; average assigned to phage 3.45%. Hereafter, sequences matched to a specific virus are referred to only by the virus name.

### Bacteriome taxonomic identification

Quality control and human reads filtering was performed on raw metagenomic reads using KneadData version 0.12.0,^51^ which involves using Trimmomatic (v 0.36),^52^ concatenating reads, and removing reads found in the human genome (version hg37dec_v0.1). Trimmomatic trimmed reads using a sliding window approach, removing reads that average <20 Phred quality score over 4 bases and keeping a minimum length of 70% of the input length. The quality-controlled reads were then run through MetaPhlAn^53^ version 4.1.1 aligning to the CHOCOPhlAn (CHOCOPhlAn_201901) database to obtain estimated bacterial counts. Default settings in MetaPhlAn were used, which include “very-sensitive” for Bowtie2,^54^ collecting all taxonomic levels, a minimum total nucleotide length for markers in a clade of 2000 (--min_cu_len), no minimum alignment length requirement, a quantile value of 0.2 (--stat_q) and 33% of markers nonzero to prevent misidentification (--perc_nonzero). An average of 23.3 million reads per sample were classified as bacteria.

### *Bifidobacterium longum* subsp. *infantis* quantitative PCR

We assessed *B. infantis* presence utilizing a *B. infantis*-specific primer set targeting the Blon_0915 region in a probe-based qPCR analysis.^55^ To derive relative abundance of *B. infantis*, universal primers that anneal to conserved regions of the 16S rRNA were also used to quantify total 16S copies using intercalator-based qPCR as previously described by us.^18^ Standard curves were prepared from serial dilutions of known cultures, and negative controls were included for each assay. Samples were analyzed in duplicate. It was assumed that 16S copy number among the overall microbiome was five and 16S copy number among *B. infantis* was four. Therefore, relative abundance was derived as [*B. infantis* species-specific gene copies]/([16S copies]/[4/5]). Infants with a relative abundance of *B. infantis* greater than 0.0000% were included in the *B. infantis* positive group.

### Statistical analysis and visualization

To reduce spurious results and remove noise, unless otherwise stated, taxa present in less than 10% of samples at each visit were discarded from further analysis. For bacteriophage analysis, any identified phage whose taxonomy (as classified by NCBI nr/nt database) terminated in “environmental samples” or “unassigned” or “unclassified” (for example, “unclassified Inoviridae”) were removed. After these steps, of the original 215 genus-level taxa, 204 remained in the 6-week visit, 203 remained in the 6-month visit, and 205 remained in the 12-month visit. Relative abundance was calculated based on reads relative to total unique, quality-controlled reads per sample. R statistical software (version 4.3.1) or GraphPad Prism (version 10.2.0) was utilized for all analyzes and visualization. Species richness and Shannon diversity (alpha diversity) were ascertained using the BiodiversityR package (version 2.17-2) in R. For three-way comparisons across time, to account for repeated measures and missing values, a mixed effects analysis was used with Geisser–Greenhouse correction. For three-way comparisons within timepoints, the Kruskal‒Wallis rank sum test was performed with Dunn's multiple comparisons test. For two-way comparisons, Wilcoxon rank sum test was performed. Heatmaps were generated on normalized reads centered and scaled using the compositions R package. Taxa relative abundance bar plots were plotted using phyloseq on mean relative abundances per sample. The alluvial diagram was created using the ggalluvial package. Barplots and line diagrams were generated using ggplot2. Core taxa were calculated per visit using the microbiome R package using a detection of 1/10,000 and prevalence of 20%. Venn diagrams showing relationships of these taxa shared between visits were plotted using the VennDiagram R package.

The Bray‒Curtis dissimilarity index, calculated using vegdist, was used to assess beta diversity, followed by PERMANOVA for statistical analysis. PERMANOVA across timepoints was performed by restricting the permutation within subjects. Contingency tables were analyzed using Fisher's exact test with unadjusted odds ratio and 95% confidence intervals calculated for each variable. To determine differentially abundant taxa at each timepoint, LinDA (MicrobiomeStat package), a linear model for differential abundance that accounts for the compositionality, was used.^56^ For each visit, data was entered as proportions and zero values were imputed by half of the minimum for each feature. The log_2_-fold change and log_-10_ *p*_*adj*_-values were graphed using ggplot2. For DA taxa with *B. infantis* presence, multivariate analysis was performed to include ROC and OOM in the calculations.

For viral network correlation analysis at each visit, we removed species absent in more than 25% of samples to reduce spurious correlations and measured correlation between species using Kendall's correlation coefficient. For bacterial-phage correlations, bacterial species present in at least 25% of samples and viral species determined to be differentially abundant while examining the OOM and ROC cohorts were used. Correlations were graphed using the ggcorrplot package. For network diagrams, taxa with correlations of adjusted *p ≤* 0.01 were plotted using the igraph package. To create animations, the ggnetwork package was used to plot and connect these networks over time. Unless otherwise stated, correction for multiple comparisons was performed using the Benjamini‒Hochberg method with significance determined by FDR ≤ 0.05.

## Results

### Infant demographics and exposures

Mother–infant dyads were recruited prenatally during the second and third trimesters from the OOM of Western New York and urban/suburban Rochester, NY (ROC) populations as part of the “Zooming in on Old Order Mennonites” (ZOOM) cohort, described elsewhere in detail.^46^ Key characteristics differentiating the OOM population include increased rates of vaginal delivery, home births, exposure to farm animals/livestock, and exclusive breastfeeding, as well as decreased rates of infant antibiotic use and pets living inside the home when compared to the ROC urban population (Table 1). Data on outcomes of allergic/atopic disease made by 2 y of age in this study are detailed in Table S1. Among ROC, 23 infants developed AD by 2 y of age, nine infants developed IgE-mediated FA (in one associated with allergic proctocolitis), and two developed allergic proctocolitis associated with other gastrointestinal symptoms which resolved; among OOM, two infants developed AD. The most common IgE-mediated FA was egg allergy (*n* = 8). The average age of symptom onset was 6.4 (range, 1–24) months. There were no cases of eosinophilic esophagitis or food protein-induced enterocolitis syndrome.

**Table 1.** Cohort analysis (6 week visit).

Characteristic	OOM (*n* = 68)	ROC (*n* = 63)	*p*-value
**Male, *n* (%)**	43 (63)	27 (43)	0.023
**Age in d, mean (SD)**	45.5 (13.9)	48.7 (20.0)	
**Delivery method (*n*, %)**			0.00043
C-section	2 (3)	15 (24)	
Vaginal	66 (97)	48 (76)	
**Delivery location**			<0.0001
Home	51 (75)	2 (3)	
Hospital	16 (24)	59 (94)	
N/A	1 (1)	2 (3)	
**Antibiotic use (*n*, %)**	2 (3)	6 (10)	0.15
**Feeding methods (*n*, %)**			
Exclusive breast feeding	60 (88)	38 (60)	0.00027
Formula supplementation	7 (10)	24 (38)	0.00020
Solid food supplementation	1 (1)	1 (2)	
**Animal exposure (*n*, %)**			
Have pets	65 (96)	51 (81)	0.012
No pets	3 (4)	12 (19)	
Cats	43 (63)	19 (30)	0.00021
Dogs	59 (87)	39 (62)	0.0013
Birds	3 (4)	5 (8)	
**Pets reside in house (*n*, %)**	11 (16)	50 (79)	<0.0001
**Farm animal exposure**			
Have exposure	67 (99)	6 (10)	<0.0001
No exposure	1 (1)	57 (90)	
Horse	62 (91)	3 (5)	<0.0001
Cow	45 (66)	2 (3)	<0.0001
Pig	25 (37)	2 (3)	<0.0001
Poultry	55 (81)	5 (8)	<0.0001
Other	22 (32)	3 (5)	<0.0001
**Average number of siblings**	2.38	1.29	0.0004

Number of participants in overall cohort for each category are indicated next to that category. For categorical variables Fisher's exact test was used to determine statistical significance (*p*-value < 0.05). For continuous variables, the Mann‒Whitney U test was used to determine statistical significance. The values that are not equal to their respective groups total contain individuals for whom there was no data available. OOM = old order mennonites; ROC = Rochester; CI = 95% confidence interval; lbs = pounds.

### The phageome diversity increases within the first year of life

Infant stool samples (68 OOM and 63 ROC infants) were processed through shotgun metagenomic sequencing and then run through the VirusSeeker pipeline.^48^ In total, 260 infant stool samples (131 samples at 6 weeks, 67 samples at 6 months and 62 samples at 12 months) underwent DNA virome analysis (Figure 1A). Consistent with previous data,^19^^,^^57^ bacteriophages predominated in the enteric virome, with *Caudoviricetes* members being the most common phages found. Similar to prior studies,^41^^,^^58-63^ bacteriophage species richness and alpha diversity significantly increased over the first year of life (*p* < 0.001, Figure 1B, 1C). We then examined phage beta diversity, a metric of dissimilarity between bacteriophage communities, and found that bacteriophage community composition significantly differed between visits (*p =* 0.001, Figure 1D). To further explore taxa composition which may be driving the bacteriophage community differences, we examined the flow of proportional abundance for all infants over the three timepoints. The pattern of bacteriophage genera proportional abundances among all infants remained similar over time (Figure 1E), with some notable difference. *Lambdavirus* (Figure 1E, dark pink) proportional abundance appeared to decrease relative to other genera between 6 weeks and 12 months of age. To examine individual infant DNA phageome changes over the first year of life, we next looked at the 53 infants (OOM *n* = 28, ROC *n* = 25) for whom we had virome data at all timepoints. The bacteriophage abundance pattern of the 40 most abundant bacteriophage genera appears to invert between 6 and 12 months in all infants regardless of lifestyle (Figure 1F), suggesting that a common factor present in both lifestyles, such as the introduction of solid food or cessation of breastfeeding, may be impacting bacteriophage genera presence. However, many similarities in the phageome exist overall between timepoints. Examining core shared taxa present within at least 20% of samples reveals that, although taxa abundances differ between timepoints (Figure 1F), the majority of genera are shared between all timepoints (Figure 1G).

### Lifestyle differences have an impact on the infant phageome

**Figure 2.**
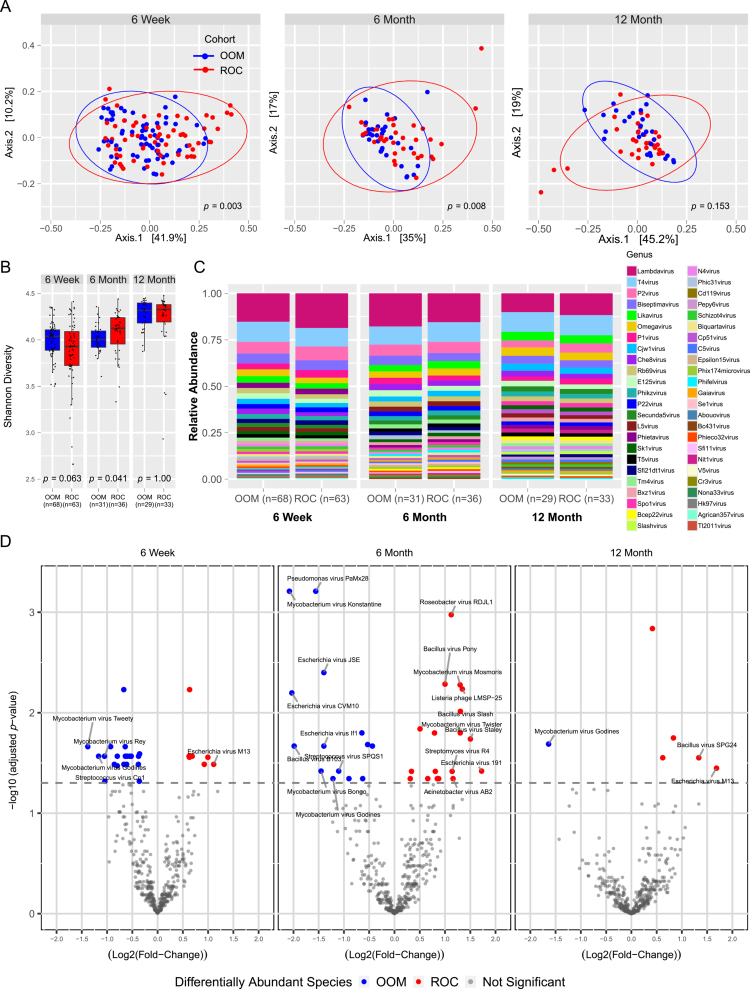
Enteric bacteriophage populations differ between OOM and ROC infants. (A) Principal coordinate analysis measured using the Bray‒Curtis dissimilarity index and (B) Shannon diversity of the different lifestyle groups at the bacteriophage genera level across the three visits. (C) Relative abundance bar graphs showing the 40 most abundant bacteriophage genera across the three visits split by cohort (OOM vs ROC). (D) Volcano plot of differentially abundant bacteriophage species between OOM and ROC. The y-axis indicates adjusted *p*-value (scaled by log_-10_), x-axis shows log_2_-fold change in relative abundance. Significant taxa, *p*_*adj*_ ≤ 0.05 (indicated by the gray dotted line), are noted in color. Taxa that have either greater than a 1 log_2_-fold change or a significance of *p*_*adj*_ ≤ 0.01 are identified with labels. OOM = blue, ROC = red. 6 weeks: OOM, *n* = 68, ROC, *n* = 63; 6 months: OOM, *n* = 31, ROC, *n* = 36; 12 months: OOM, *n* = 29, ROC, *n* = 33.

Since living in single-family farming communities is associated with decreased incidence of atopic disease in this study (Table S1) and others,^17^ and a cross-sectional study of this population showed changes in the bacteriome,^18^ we examined the phageome between the ROC urban/suburban lifestyle infants and single-family traditional farming lifestyle OOM infants (Figure 2). Bacteriophage population-level differences between the two lifestyles were examined using Bray–Curtis dissimilarity index (Figure 2A), showing bacteriophage communities significantly differed between ROC and OOM infants at 6 weeks and 6 months (*p* = 0.001). Shannon diversity, was significantly higher at 6 months in the ROC cohort (*p* = 0.0405), with a trend towards lower diversity in the ROC cohort at 6 weeks (*p* = 0.0631; Figure 2B). By 12 months, there was no difference in alpha or beta diversity between the two cohorts, suggesting that phage diversity differences may be more affected by lifestyle in early infancy than in later infancy. We then assessed core viral taxa and found that the majority of core phage taxa were shared between ROC and OOM (189 out of 199). Species richness did not differ at any timepoint either (data not shown). Bacteriophage genera relative abundances among the most abundant taxa were assessed and revealed similar patterns between the two lifestyles at all visits (Figure 2C). We also found shared patterns of fluctuations in abundant phage populations over time (Figure S1) regardless of lifestyle groups. These data highlight the similarities between the core phageomes in both lifestyle groups despite differences in environmental exposures. Since overall bacteriophage communities differed as determined by beta diversity, these results suggest that phageome community differences between lifestyles are due to rare or less abundant species and their distribution.

We then assessed for differentially abundant (DA) bacteriophage species between OOM and ROC infants (Figure 2D and Table S2). There were 27 DA phage species at 6 weeks, 32 at 6 months and six species at 12 months. *Mycobacterium virus Godines* was more abundant in OOM at all timepoints, revealing bacteriophage species persistence over the first year of life. Most of the known *Mycobacterium* phages were originally isolated from soil and compost.^64^^,^^65^ Farming practices in the OOM group may increase exposure to soil bacteria and therefore aid in colonization with corresponding phages. No *Mycobacterium* viruses were more abundant in ROC at 6 weeks or 12 months. *Escherichia virus M13* was significantly more abundant at 6 weeks and 12 months in the ROC group, while *Escherichia virus E112* was more abundant in OOM at 6 weeks and 6 months. These are phages which infect more common gut bacteria. *Escherichia virus M13* is a lysogenic filamentous phage and was initially isolated from human sewage,^66^ denoting its prevalence in urban environments. *Bacillus* viruses were more prevalent in the ROC group at all timepoints, with *Bacillus virus pony* significantly more abundant in ROC infants at both 6 weeks and 6 months (Figure 2D and Table S2). These data again are consistent with lifestyle differences influencing the enteric phageome in the first 6 months of life.

Delivery mode differed between the cohorts (Table 1), and this is a known confounder for the bacterial microbiome composition and stability.^67^ To evaluate if delivery mode impacted the virome in this cohort, we assessed for viral community similarity between infants born by vaginal and cesarean method in all samples irrespective of lifestyle group at 6 weeks and 6 months by PERMANOVA and found no difference in virome beta diversity at 6 weeks (*p* = 0.860) or 6 months (*p =* 0.876), suggesting that delivery mode overall had little impact on the virome at these time points. It is likely that earlier timepoints would have shown a greater impact of delivery mode if they had been available.^67^

### The bacteriome is dominated by *Bifidobacteriaceae*

**Figure 3.**
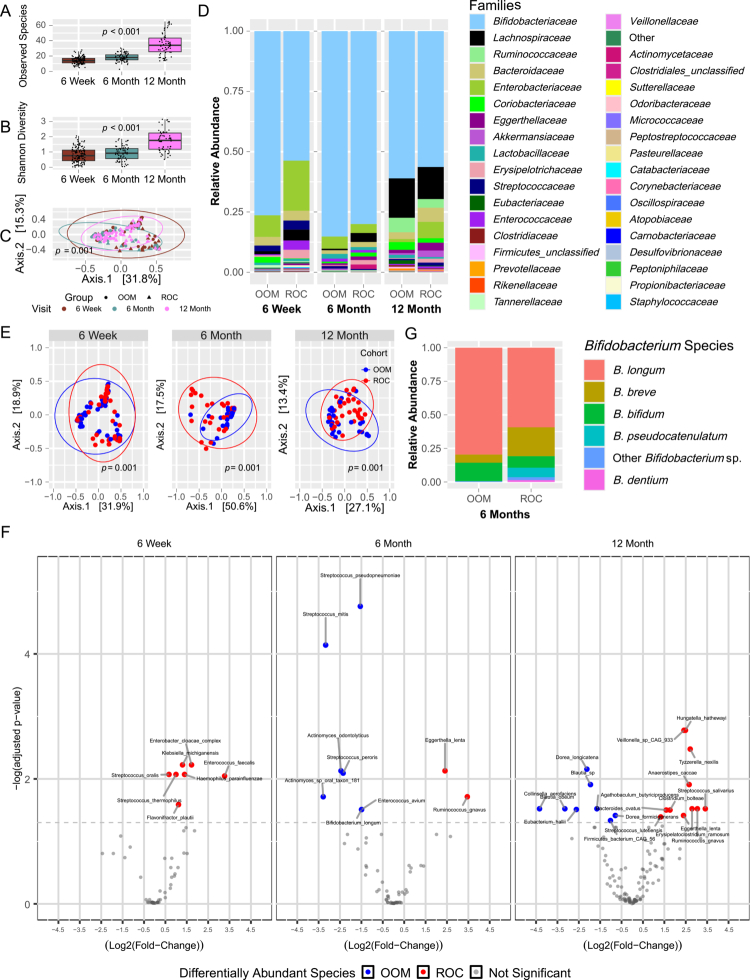
Enteric bacteriome composition differs between ROC and OOM. (A) Total bacterial species count and (B) bacterial species Shannon diversity of all subjects at each visit. (C) Principal coordinate analysis of all samples across the three visits as measured by the Bray‒Curtis dissimilarity index. Circles = OOM, triangles = ROC. (D) Relative abundance bar graphs showing bacterial families across the three visits split by lifestyle (OOM vs ROC). “Other” represents identified species that were classified as bacterial without family-level phylogenetic identification. (E) Principal coordinate analysis measured using the Bray‒Curtis dissimilarity index of the different lifestyle groups at the species level across the three visits. (F) Volcano plot of differentially abundant bacterial species between OOM and ROC. The y-axis indicates adjusted *p*-value (scaled by log_-10_), x-axis shows log_2_-fold change in relative abundance. Significant taxa, *p*_*adj*_ ≤ 0.05 (indicated by the gray dotted line), are noted in color. Taxa that have either greater than a 1 log_2_-fold change or a significance of *p*_*adj*_ ≤ 0.01 are identified with labels. OOM = blue, ROC = red. 6 weeks: OOM, *n* = 68, ROC, *n* = 63; 6 months: OOM, *n* = 31, ROC, *n* = 36; 12 months: OOM, *n* = 29, ROC, *n* = 33. (G) Relative abundance bar graph of *Bifidobacterium* sp. at 6 months, grouped by lifestyle cohort.

We next ascertained the bacterial microbiome (bacteriome) by running the metagenomic sequencing data through KneadData^51^ and MetaPhlAn4.^53^ Similar to the virome, richness and Shannon diversity of the bacteriome increased over time (Figure 3A and B), and bacterial communities significantly differed between timepoints (Figure 3C). Bacterial species richness greatly increased between the 6-month and 12-month timepoint, likely due to increasing environmental and dietary exposures. Family-level analysis showed that the bacteriome was dominated by *Bifidobacteriaceae* (Figure 3D) in both ROC and OOM infants at all timepoints. *Bifidobacteriaceae* was significantly more abundant in OOM at six weeks (*p* = 0.001), while *Enterobacteriaceae* was more abundant in ROC at six weeks (*p* = 0.026). Bacterial communities were also significantly different between lifestyle groups at all timepoints (Figure 3E). Similar to the virome, bacteriome species richness did not differ between ROC and OOM; however, unlike the virome, Shannon diversity showed no significant differences between lifestyle groups at any timepoint (ROC vs OOM, Figure S2 A, B).

We then assessed differentially abundant taxa between lifestyle groups (Figure 3F, Table S3). Seven bacterial taxa were differentially abundant at 6 weeks, all at higher levels in ROC infants, nine at 6 months, and 19 at 12 months. Notably, *B. longum* was more abundant in OOM than ROC at 6 months. To more closely examine the contribution of different *Bifidobacteraceae* taxa, relative abundances of the identified species were graphed (Figure 3G) at 6 months, revealing that *B. longum* is the most abundant of the *Bifidobacterium* species. Unfortunately, we were unable to resolve subspecies using MetaPhlAn4 for taxonomic identification. These data overall show that lifestyle factors significantly result in distinct bacterial communities, with *B. longum* subspecies an important component of the bacterial microbiome in both lifestyle groups.

### The phageome differs with *Bifidobacterium longum* subsp*. infantis* (*B. infantis*) presence

**Figure 4.**
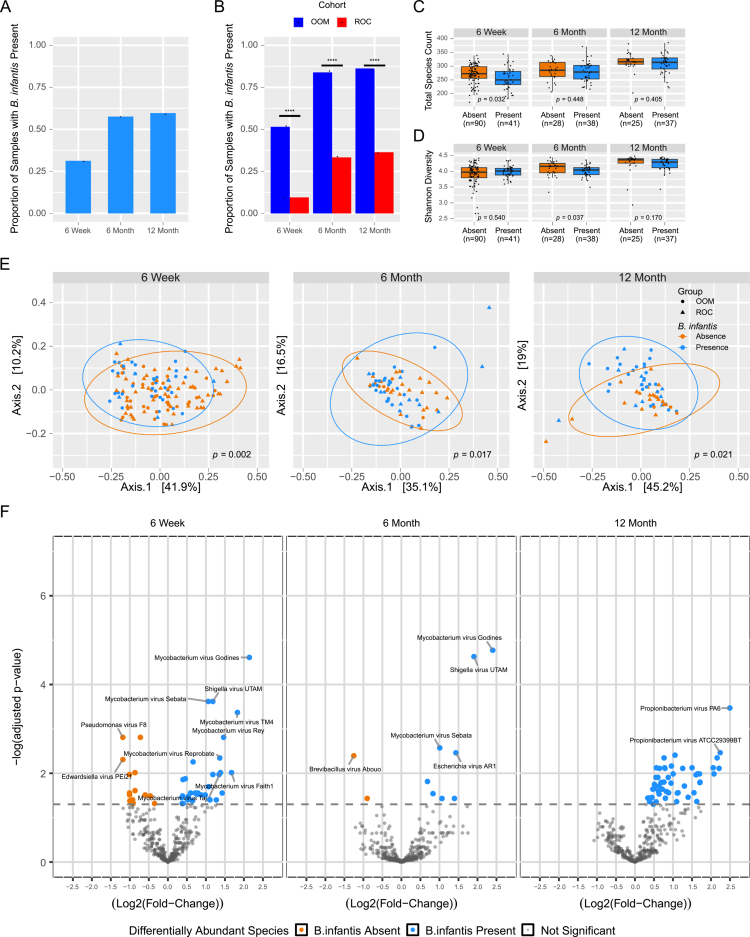
The early infant enteric phageome differs in the presence of *B. infantis*. (A) Proportion of stool samples with *B. infantis* presence (y-axis) at each timepoint (x-axis) as determined by qPCR. (B) Proportion of stool samples with *B. infantis* presence (y-axis) at each timepoint (x-axis) separated by lifestyle group (OOM, blue; ROC, red). (C) Number of bacteriophage species and (D) bacteriophage genus-level Shannon diversity of all subjects by *B. infantis* presence or absence across the three visits. (E) Principal coordinate analysis of bacteriophage communities colored by *B. infantis* presence/absence at each collection time measured using Bray–Curtis dissimilarity index. (F) Volcano plot of differentially abundant bacteriophage species by *B. infantis* presence/absence, with multivariate analysis accounting for lifestyle groups. Y-axis indicates adjusted *p*-value (scaled by log_-10_), x-axis shows log_2_-fold change. Significant bacteriophage taxa, *p*_*adj*_ ≤ 0.05 (indicated by the gray dotted line), are noted in color. Taxa that have both greater than a 1 log_2_-fold change and a significance of *p*_*adj*_ ≤ 0.01 or less are identified with labels. Blue = *B. infantis* present in stool samples; Orange = *B. infantis* not found.

*Bifidobacterium longum* consists of three subspecies *– longum, infantis,* and *suis. While B. longum* subsp *longum* is identified in both adults and infants,^68^ *B. longum* subsp*. infantis* (*B. infantis*) is a keystone bacteria enriched in breastfed infants, particularly those in nonindustrialized communities at low risk for atopic disease.^68^ *B. infantis* is unique in its ability to efficiently utilize all human milk oligosaccharide,^18^^,^^69^ (Henrick et al., 2021; Seppo et al., 2021a) giving it a distinctive ecological niche for the duration of breastfeeding. Depletion of *B. infantis* has been associated with increased systemic inflammation and immune dysregulation, which may predispose to atopic disease.^70^^,^^71^ We have previously shown a high abundance of *B. infantis* in the OOM community.^18^ MetaPhlAn, our current method of determining bacteriome taxonomy, cannot differentiate between *B. longum* subspecies *longum* and *infantis,* both of which can dominate early infant bacteriome. In order to differentiate between the two, and to further assess the relationship between the phageome and bacterial communities, qPCR specific for *B. infantis* was performed on infant stool samples. Since *B. infantis* tends to dominate infant bacteriome in a transient pattern when present in breastfed infants, samples were simply designated *B. infantis* positive or negative irrespective of the qPCR determined relative quantity, and the phageome was assessed in these two groups (Figure 4). The proportion of samples with *B. infantis* colonization increased from 6 weeks to 6 months, then stabilized (Figure 4A). A significantly higher proportion of infants in the OOM cohort had *B. infantis* in their gut microbiomes present at all timepoints (Figure 4B), with *B. infantis* presence 10 times more likely than absence in the OOM group at all timepoints (*p* < 0.0001; odds ratio (OR) 6 weeks 10.08 (confidence interval (CI): 3.726–25.51), 6 months OR 10.4 (CI: 3.029–30.29), 12 months OR 10.94 (CI: 3.19–33.17)). These data show that while *B. longum* is highly abundant in both ROC and OOM (Figure 3G), the *B infantis* subspecies is particularly enriched in single-family farming lifestyles.

To examine the phageome in the context of enteric *B. infantis* colonization, we assessed phage richness and diversity. Phage species richness was significantly lower at 6 weeks (Figure 4C), but phage Shannon diversity was significantly lower at 6 months in infants with *B. infantis* present (*p* = 0.037; Figure 4D). These data cannot differentiate whether phageome differences were more impacted by *B. infantis* colonization or lifestyle group at this timepoint. However, they suggest profound differences in phageome populations in the presence of this dominant keystone bacterial species. We also found that the phageome community structure was dissimilar throughout the first year of life between infants with or without *B. infantis* colonization (Figure 4E). Notably, no *Bifidobacteriaceae-*infecting bacteriophages were found in our analysis, suggesting indirect effects of *B. infantis* on the phageome. To identify individual phage taxa contributing to this effect, we assessed DA taxa at each timepoint (Figure 4F). Since there were significant differences in the presence of *B. infantis* between lifestyle groups (Figure 4B), we performed multivariate analysis. We found 46 species enriched by *B. infantis* presence or absence at 6 weeks, 10 DA species at 6 months, and 54 DA species at 12 months (Table S4), accounting for lifestyle differences. *Mycobacterium* phages represented 17 of 31 phage species significantly more abundant in infants colonized with *B. infantis* at 6 weeks, and *Mycobacterium* phages predominated at the later timepoints as well. *Mycobacterium virus Godines* was markedly more abundant in infants colonized with *B. infantis* at all 6 weeks and 6 months, independent of lifestyle group, making this phage of interest for future studies. On the contrary, *Shigella*, *Escherichia*, and *Bacillus* phages predominated in infants lacking *B. infantis* at all timepoints (Figure 4F, Table S4). Overall, these data suggest that while richness and evenness of species is similar, the underlying bacteriophage community composition is shaped by *B. infantis* colonization in the first year of life.

### The early life phageome composition trends toward association with subsequent atopic outcomes

**Figure 5.**
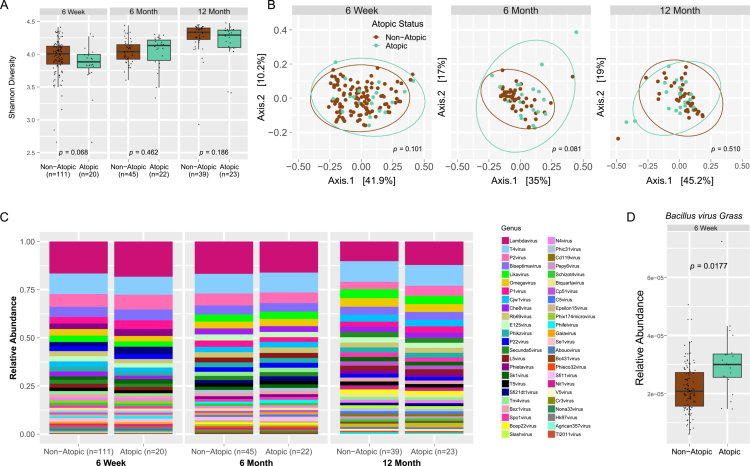
Early life enteric bacteriophage populations between atopic and nonatopic infants. (A) Shannon diversity and (B) Principal coordinate analysis as measured using the Bray‒Curtis dissimilarity index comparing bacteriophage populations in atopic versus nonatopic infants at the genera level across the three visits. (C) Relative abundance bar graphs showing the 40 most abundant bacteriophage genera across the three visits split by atopic status. (D) Boxplot of the only DA bacteriophage taxa by atopic status, *Bacillus virus Grass*, at 6 weeks. Nonatopic = brown, atopic = aquamarine; 6 weeks: nonatopic, *n* = 111, atopic, *n* = 20; 6 months: nonatopic, *n* = 45, atopic, *n* = 22; 12 months: nonatopic, *n* = 39, atopic, *n* = 23.

To examine whether the infant enteric phageome may impact later development of atopic disease, we examined the enteric phageome in infants who later developed atopic disease (FA and/or AD) by 2 y follow-up (Figure 5; Table S5). To quantify whether the phageome differed between atopic and nonatopic infants, we first assessed species richness, and alpha and beta diversity of the enteric bacteriophages. We found no significant differences in species richness (data not shown); however, there was a trend toward higher Shannon diversity in phageomes of nonatopic infants at 6 weeks (*p* = 0.068; Figure 5A) and a trend toward altered phageome communities as assessed by beta diversity at 6 months (*p* = 0.081; Figure 5B). The relative abundances of the enteric bacteriophage genera between the two groups appeared similar between the nonatopic and atopic groups at all timepoints (Figure 5C), with the three most abundant taxa remaining as *Lambdavirus*, *T4virus*, and *P2virus* at all timepoints. Analysis for DA taxa by atopic status showed *Bacillus virus Grass* was significantly more abundant at 6 weeks in infants who later developed atopic disease (*p*_*adj*_ = 0.0177, Figure 5D and Table S6), but no other DA taxa were found at other timepoints. Though underpowered to conclusively ascertain associations between the infant virome and atopic disease, these data support the need for further research into early (≤6 months) enteric bacteriophage populations in infants that develop atopic disease.

Since most infants that developed atopic disease belonged to the ROC group, we wondered whether the trend toward phageome differences found in infants who later developed atopic disease might reflect lifestyle differences between the ROC and OOM groups. While there were too few atopic infants to perform multivariate modeling, to remove the influence of lifestyle and potential genetic differences in this comparison, we repeated the above analysis on ROC cohort infants only (Figure S3). Bacteriophage genera relative abundance (Figure S3A), bacteriophage species richness (Figure S3B), Shannon diversity (Figure S3C), and beta-diversity (Figure S3D) were similar between atopic and nonatopic infants in the ROC cohort. Because low numbers of individuals with virome data and atopic disease limits power to detect differences, we cannot rule out that lifestyle differences may have contributed to the differences in phageome composition in atopic infants.

### Enteric bacteriophage-bacteriophage correlations change over the first year of life

We next examined enteric bacteriophage-bacteriophage interactions over time during infancy. Data from phage therapy trials testing cocktails of phages to combat resistant bacteria has shown that efficacy of phage combinations often does not equal the sum of the component phages' efficacy.^72^^,^^73^ This is due to interactions between phages, whether cooperative or antagonistic. Phage gene function can depend on the presence of unrelated bacteriophages, such as the requirement for phage coinfection by a helper phage to mobilize genetic elements, encode the receptor for another phage, and toxin subunits encoded on different phages.^72^ Recent work has also shown that some phages encode quorum sensing systems to regulate phage-induced lysis in nearby bacteria,^74^ demonstrating another mechanism for phage–phage communication and interaction.

**Figure 6.**
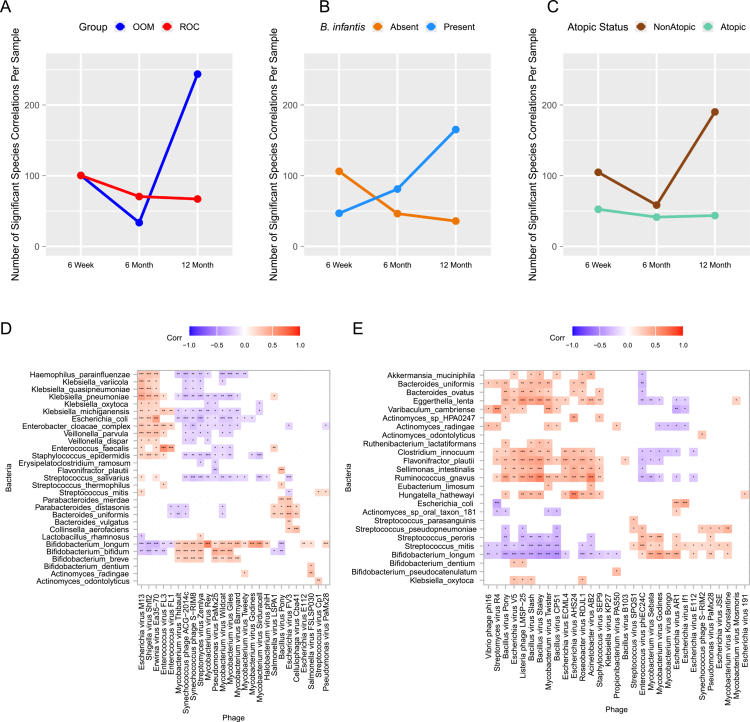
Enteric phage-phage and phage-bacterial correlations across the first year of life. Correlations between bacteriophage species present in at least 25% of samples at each visit were determined by Kendall's correlation coefficient. Average significant phage-phage correlations, adjusted for multiple comparisons, per sample (y-axis) were plotted over the three timepoints grouped by (A) lifestyle, (B) *B. infantis* presence or absence, and (C) atopic status. (D, E) Bacterial species present in at least 25% of samples were correlated to DA viral species between ROC and OOM using Kendall's correlation coefficient. Correlation plots of significant phage-bacterial associations adjusted for multiple comparisons at (D) 6 weeks and (E) 6 months. Color, as shown in the key.

Kendall's correlation analysis was used to determine positive and negative phage-phage associations, adjusted for multiple comparisons, and significant phage species associations were plotted across the three timepoints. Numerous significant associations were found at each timepoint. To more easily quantify these differences over time, we graphed the number of significant phage-phage species correlations per sample over all timepoints for each comparator group (Figure 6). For most viral comparisons, 6 months appears to be a pivot point, possibly due to dietary expansion, greater colonization by *B. infantis*, or other exposures. The number of significant phage-phage correlations between ROC and OOM lifestyle is similar at 6 weeks, but by 1 y of age the lifestyle groups diverge, with the OOM group having thrice as many significant phage-phage correlations per sample (Figure 6A). Infants without enteric *B. infantis* colonization initially started with higher numbers of significant phage-phage interactions at 6 weeks, but this number decreased substantially by 6 months, whereas associations steadily increased in infants with enteric *B. infantis* colonization (Figure 6B). This may be impacted by the higher proportion of infants having *B. infantis* in their gut microbiomes at 6 and 12 months (Figure 4A), though a similar trend was seen when samples were evenly subsampled over 100 iterations (data not shown). Phage-phage correlation plots comparing lifestyle groups graphically demonstrate these data (Figure S4).

To further visualize how bacteriophages interact with each other over time, we performed phageome network analysis (Figures S5 and Video1). Due to the excessive number of phage-phage associations, we narrowed to core phages present in at least 25% of samples and plotted those significantly different after multiple comparisons adjustment (*p*_*adj*_ < 0.01). Six months appears to be a pivot point in phageome networks in both ROC and OOM (Figure S5A), seen easily in the animation (Video1). While ROC and OOM both start at 6 weeks with dense nodal confluences, by 6 months there are fewer phage nodes and few negative phage-phage associations. OOM returns to a dense nodal confluence at 12 months, and ROC exhibits a sparser network with a higher ratio of negative phage-phage associations present (Figure S5A, Video1). When we examine phageome networks between infants with and without enteric *B. infantis* colonization, networks differ at all timepoints (Figure S5B). The sparsity of strong interactions in *B. infantis* colonized infants at 6 weeks begins to pivot at 6 months and reverses by 12 months (Figure S5B, Video1). These results suggest that early exposure to urban lifestyles and absence of *B. infantis* colonization in infancy may lead to profound lasting alterations in the infant gut DNA phageome that multiplies over time.

### Bacteriophage-bacteriophage networks are disrupted at all timepoints in infants who develop atopic disease

We next examined phage-phage associations by atopic status. Fewer phage-phage associations were found in infants who later develop atopic disease than in nonatopic infants at all timepoints (Figure 6C, Figure S4C), suggesting that early enteric phage-phage interactions may protect from atopic disease development. While sample size disparity might explain correlation differences observed in the 6-week timepoint (111 nonatopic vs 20 atopic), the numbers are more evenly matched in 6-month (45 nonatopic vs 22 atopic) and 12-month timepoints (39 nonatopic vs 23 atopic) despite discrepant correlation patterns, suggesting a fundamental difference in the phageomes between these infants at 6 and 12 months. Furthermore, a similar trend was seen when samples were evenly subsampled over 100 iterations (data not shown).

Phageome networks also differ at all timepoints when comparing nonatopic infants to infants who developed atopic disease (Figure S5C, Video1). Atopic infants' data show no large phage nodes or nodal confluences at any timepoint, suggesting abnormal phage-phage interactions or phageome dysbiosis. Since beta diversity did not differ between atopic and healthy infants (Figure 5B), this suggests that this sparsity is not due to bacteriophage dysbiosis but instead due to dissimilarities in interacting phage species. These data further suggests that disruption of phage-phage networks in early infancy may not be easily corrected and may contribute to atopic disease development.

### Enteric bacteriophages correlate with bacterial species

To ascertain putative direct and indirect interactions between infant enteric bacteriophages and bacterial species, we examined bacterial species correlations with DA phage taxa at 6 weeks (Figure 6D) and 6 months (Figure 6E). Significant correlations, adjusted for multiple comparisons, showed *B. longum* is a key player in phage-bacterial correlations at both timepoints. Bacteriophage correlations cluster with specific bacteria, with bacterial-bacteriophage correlation clusters at 6 weeks being dominated by gram-negative bacteria, while clusters at 6 months were dominated by gram-positive bacteria. At 6 weeks (Figure 6D), phages that negatively correlated with *Bifidobacterium* species abundance, such as *Escherichia virus M13, Shigella Virus Shfl2, Enterococcus virus FL3* and *FL1,* and *Erwinia virus Ea35-70,* positively correlated with abundance of *Klebsiella* species, *E. coli, E. cloacae, Veillonella species, H. parainfluenzae* and *Enterococcus faecalis*, all of which can be associated with enteric inflammation and disease.^75^^,^^76^ Conversely, many of the *Mycobacterium* phages exhibited positive correlations with *Bifidobacterium* species abundances and negative correlations with *Streptococcus salivarius*, *Staphylococcus epidermidis, Veillonella* species, *E. cloacae, E. coli, Klebsiella* species, and *H. parainfluenzae*. Similar patterns emerge at 6 months (Figure 6E), with *Mycobacterium* viruses positively correlating with *B. longum.* At 6 months, *Bacillus* viruses show negative correlations with *Bifidobacterium* species, suggesting a less beneficial enteric microbiome in the presence of these viruses. Further studies into the mechanism behind and consequences of these correlations are warranted.

### The eukaryotic virome in infancy

**Figure 7.**
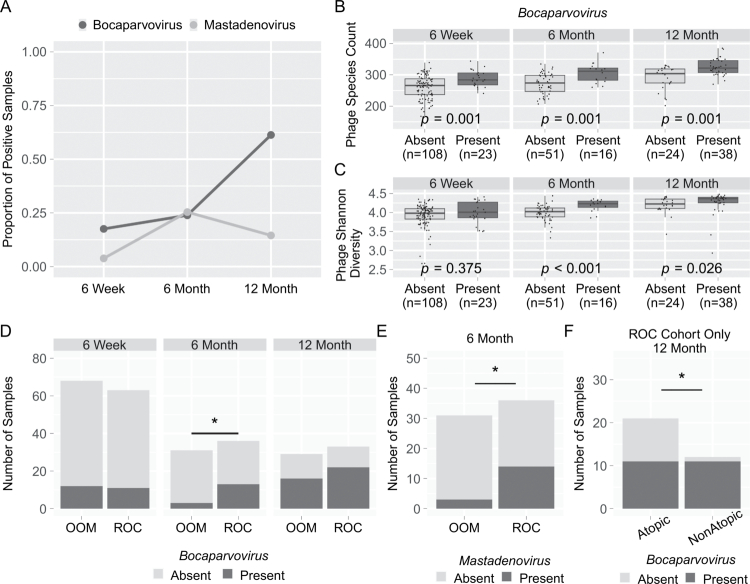
Enteric eukaryotic viruses during infancy are associated with the phageome, lifestyle and atopic disease. (A) Proportion of infant stool samples positive for *Bocaparvovirus* (blue) or *Mastadenovirus* (orange) at each timepoint. The table below indicates the percentage. (B) Bacteriophage species richness and (C) Shannon diversity in infants with *Bocaparvovirus* present in stool samples. (D) Number of samples (y-axis) with *Bocaparvovirus* present (black) or absent (gray) at each timepoint grouped by lifestyle. (E) Number of samples (y-axis) with *Mastadenovirus* present (black) or absent (gray) at 6 months in the ROC or OOM groups. (F) Number of samples (y-axis) with *Bocaparvovirus* present (black) or absent (gray) at 12 months in ROC infants who later developed atopic disease versus ROC nonatopic infants. * indicates *p* < 0.05.

We finally turned our attention to the eukaryotic virome in the first year of life. After removal of low abundance taxa (present in <10% of samples), only two viral families remained, *Adenoviridae*, comprised of *Mastadenovirus* sequences, and *Parvoviridae*, comprised mostly of *Bocaparvovirus* sequences*. Bocaparvirus* sequences were noted in 17.6% of samples at 6 weeks (*n* = 23) and increased over the first year of life to 61.3% by 12 months of age (*n* = 38; Figure 7A). To ascertain the relationship between bacteriophage populations and *Bocaparvovirus* presence, we assessed bacteriophage richness (Figure 7B) and alpha diversity (Figure 7C) grouped by *Bocaparvovirus* presence/absence. Samples where *Bocaparvovirus* was present showed significantly higher phage species richness at all timepoints (*p* = 0.001) and higher diversity at 6 (*p* < 0.001) and 12 months (*p* = 0.026). We then evaluated the impact of lifestyle differences on *Bocaparvovirus* presence (Figure 7D) across the first year of life, and found similar levels at 6 weeks, but significantly higher rate of positivity in the ROC cohort at 6 months (*p* = 0.0198, OR 5.275 (CI: 1.353–18.62)). This may represent differences in lifestyle-associated exposures such as daycare which is limited to the ROC cohort.^46^ Interestingly, there is no significant difference in enteric *Bocaparvovirus* incidence in infants between lifestyle groups at 12 months of age (*p* = 0.4365, Figure 7D), though the overall prevalence was higher (Figure 7A). *Mastadenovirus* was found in only 3.8% of samples (*n* = 5) at 6 weeks, increasing to 25.4% at 6 months (*n* = 17) and down to 14.5% by 12 months (*n* = 9; Figure 7A). Due to low rates of positivity, *Mastadenovirus* was further analyzed only at the 6-month timepoint. There was no association between *Mastadenovirus* presence and phage richness or diversity at 6 months (data not shown), but viral sequences were significantly more prevalent in the ROC cohort than OOM at 6 months of age (Figure 7E; *p* = 0.0101, OR 5.939 (CI 1.552-20.84)).

To further evaluate whether there was a relationship between eukaryotic viral sequence presence and the development of atopic disease, we focused on the ROC cohort (to control for lifestyle exposure differences) and on *Bocaparvovirus* at 12 months due to highest numbers of positive samples at that timepoint (Figure 7F). ROC infants who developed atopic disease by 2 y of age were 10-times less likely to have *Bocaparvovirus* sequences at 12 months of age (*p* = 0.0273, OR 0.1 (CI: 8.54e10^−4^ −0.7421)). While this study was not powered to detect differences at other timepoints, this finding may suggest a protective effect of exposure to specific viruses in later infancy. Alternatively, since early manifestations of atopic disease occur prior to 12 months, this could reflect differences in the immune response to viral infection. These results are worth pursuing in future studies.

## Discussion

In this study, we characterized the infant enteric virome from 131 single family farming and urban/suburban infants with disparate risk for atopic disease development over the first year of life. Although there are many similarities in core species, the infant gut phageome also differs between lifestyle groups. For the first time we show that the infant enteric phageome diversity differs in early life between farm-life and urban/suburban lifestyle groups, with soil-dwelling *Mycobacterium* phages more abundant in farm-life infants. This study adds to the scant literature examining the infant virome,^19^^,^^58-63^^,^^77-80^ showing that the infant enteric phageome shifts between 6 and 12 months of age, likely due factors such as introduction of solid foods and increased environmental exposures in the setting of increasing infant mobility. We identified an increase in both enteric bacterial and bacteriophage richness and diversity and eukaryotic virus colonization across timepoints. These data also reveal novel insights into interactions between and within microbiome components in the infant gut, including phage-bacteria and phage-phage interactions. We showed that the phageome differs in infants with *B. infantis* colonization, suggesting key differences in infant enteric microbial communities associated with *B. infantis* presence.

While bacteriophage can alter bacteriome composition,^19^ the bacteriome can also indirectly impact the phageome communities. This is demonstrated by phageome community differences in infants colonized by *B. infantis* at 6 weeks and 6 months, although no known *Bifidobacterium* phages were found at any timepoint. Phage-bacteria correlation plots revealed that bacteriophages that positively correlated with *B. longum*negatively correlated with bacteria associated with higher gut inflammation. Interestingly, at the earliest timepoint, 6 weeks, gram-negative bacteria containing LPS in their cell membrane were more likely to be present in clusters with opposing correlations to the cluster containing *B. longum.* This suggests a model in which *B. infantis* promotes a gut microbiome colonized by more beneficial bacteria and bacteriophages. One mechanism behind this could be protection from colonic mucin barrier degradation by *B. infantis*, ^81^ leading to decreased enteric inflammation and decreased LPS translocation.

Significantly more infants developed atopic disease in the ROC than OOM communities in this study,^46^ allowing comparison of the enteric virome composition between those who did or did not develop early atopic disease. Bacteriophage communities were dissimilar at 6 weeks and 6 months of life between single family farming and urban/suburban lifestyles, suggesting that the infant phageome is associated with atopic disease risk. Since the bacteriome differed between ROC and OOM at 2 months in our prior cross-sectional study of this population,^18^ differences in the phageome are not unexpected. We found differentially abundant phage species including *Mycobacterium* phages higher in OOM while *Escherichia, Enterococcus* and *Bacillus* phages were elevated in ROC. *Mycobacterium* phages also positively correlated with *B. longum* abundance at 6 months, supporting a model where these phages are present in an enteric milieu that is protective against development of atopic disease. Bacterial analysis showed that *Bifidobacteriaceae* predominated in both lifestyle groups. This concurs with our cross-sectional study in this same population that found that OOM infant stools at 2 months of age were enriched in *Bifidobacteriaceae* and *Actinomycetia* (formerly *Actinobacteria*) class among others, while ROC stools were enriched in *Lactobacillaceae*.^18^ *Mycobacteria* species serve as hosts to the various *Mycobacterium* phages that were more abundant in OOM infant stools at all timepoints and are members of the *Actinomycetia* class, further supporting these findings. Our bacteriome analysis found *Actinomycetia* members in this cohort, but they were greatly overshadowed by *Bifidobacteriaceae*. However, no *Mycobacterium* bacterial species were identified. It is possible that these bacteria were below the limit of detection of our bacterial taxonomic identification pipeline. It is also possible that the sequences recognized by our pipeline as *Mycobacterium* phages were misidentified. Similar to our prior data,^18^ we found that enteric *B. infantis* colonization was more common in OOM than ROC. Co-occurrence networks of 2-month-old infants from our prior cross-sectional study in this population showed *Bifidobacterium* as one of the most central bacteriome nodes in OOM, with reduced centrality in ROC infants.^18^ It is therefore not surprising that phage-phage correlation networks differed in infants colonized with *B. infantis* in our study. These data highlight the influence of this keystone bacterial species on the early infant gut bacterial and bacteriophage populations, and suggests that the early infant enteric DNA virome develops differently in farming and urban lifestyles over the first year of life.

We found few eukaryotic viruses within the stool of infants in the first year of life. While the sequencing depth likely limited our ability to detect eukaryotic virus sequences, a high rate of human milk consumption in our study could also be a factor. A prior study found few to no enteric eukaryotic viruses in exclusively breastfed or mixed human milk/formula fed infants up to 4 months of age; only in exclusively formula-fed infants did they see infant gut eukaryotic viruses,^58^ consistent with known antiviral effects of human milk.^19^ No infants in our study were exclusively formula fed, which may also explain the low abundance of eukaryotic viruses at 6 weeks and 6 months and potentially explain the increase in *Bocaparvoviruses* found at 12 months, when human milk consumption decreases. Despite the few eukaryotic viruses found, we detected increased rates of enteric *Bocaparvovirus* and *Mastadenovirus* in urban compared to OOM infants at 6 months, which may be due to daycare attendance or other differences in environmental exposures.

Early life eukaryotic respiratory viral infections^19^ and enteric bacteriophages have been linked to onset of childhood asthma,^45^ but no prior study has examined the impact of the enteric virome on development of the earliest manifestations of atopic disease, including AD and FA, throughout the first year of life. Herein, we show significant differences in the enteric DNA phageome communities at 6 weeks and 6 months of age in infants at risk for later development of FA/AD. There are several possible mechanisms that could account for the influence of the early enteric virome on the development of atopic disease. First, the infant gut bacteriome has been shown to be key in development of atopic disease,^82^ and bacteriophages may play a role by modulating the gut bacterial populations. Early use of antibiotics is associated with increased risk of atopic disease presumably due to perturbations in the gut bacteriome,^19^ and it is plausible that early introduction of lytic bacteriophages could have a similar impact. However, bacteriophages may have a broader impact than that suggested by their bacterial host range. Murine studies have shown that phage predation can lead to cascading effects on non-host bacterial species through bacteria-bacteria interactions, leading to alterations in the gut metabolome and therefore may impact the as yet unidentified microbial metabolites which play a role in atopy development. Second, enteric viruses also have direct impacts on immune system maturation. Murine studies have demonstrated the essential role of the bacteriome on early innate and adaptive immune system development and maturation,^83^ and data from germ-free and antibiotic treated mice show that early and asymptomatic enteric eukaryotic viral infection elicits a similar response as bacteria on immune system maturation.^84-86^ Interestingly, murine studies have also shown that enteric eukaryotic virus infections in a strain-specific manner triggered a Th1-mediated loss of oral tolerance to dietary antigens in celiac disease^87^^,^^88^ and induction of systemic IgE,^89^ suggesting a possible contribution of enteric eukaryotic viral infections to FA. Bacteriophages can also directly stimulate pro- or anti-inflammatory immune responses independent of their bacterial hosts by activation of pattern recognition receptors both locally and systemically via intestinal translocation,^90^^,^^91^ which may impact development of immune tolerance.

Our data also reveal a significant association between enteric *Bocaparvovirus* presence at 12 months and protection from atopic disease development. Bocaparvoviruses are spread via fecal-oral route or respiratory droplets and are known to cause severe upper and lower respiratory tract and gastrointestinal illnesses in young children.^92-94^ Childhood respiratory infection with human bocavirus (HBoV) has been associated with recurrent wheeze and asthma, with one study finding that HBoV1 bronchiolitis during the first 24 months of life significantly increased the risk of early childhood asthma, with a higher odds ratio than RSV infection.^95^ Respiratory tract infection by human bocaparvoviruses leads to disease with pyroptosis, death of infected airway epithelial cells mediated by the inflammasome, with resulting localized inflammation,^96^ which may promote later development of asthma. Surprisingly, we found increased rates of enteric *Bocaparvovirus*in infants who did not develop atopic disease, suggesting a different outcome with enteric infection than that seen in respiratory tract infection. Different strains of HBoV have been found in the GI tract compared to respiratory strains and have frequently been reported with coinfections with other intestinal pathogens in up to 77.6% of children,^97^ suggesting that these strains may not be pathogenic in the GI tract.^98^ Thus these strains could be indicative of a commensal enteric colonization, leading to immune tolerance and protection against later development of FA or AD. Alternatively, enteric colonization by *Bocaparvovirus* could be a biomarker for a more protective gut microbial community. Enteric bacteriophage species richness and diversity were increased in infants with enteric *Bocaparvovirus* infection throughout the first year of life, suggesting a shared mechanism in the infant gut controlling both intestinal phage and eukaryotic viral populations. This mechanism could be related to host immune factors, differences in intake such as human milk, or could represent an interaction with bacterial microbiome members. Studies investigating the bacterial microbiome and host immune factors in this cohort are underway.

We also examined bacteriophage-bacteriophage correlation networks. While bacterial-bacteriophage interactions are well known, phage-phage interactions are understudied, though cooperative and antagonistic gene product interactions between unrelated bacteriophages are documented.^72^^,^^74^ Further evidence of the importance of phage-phage interactions comes from antimicrobial phage therapy studies utilizing cocktails of bacteriophages. Phage therapy is a treatment method using lytic phages to directly kill antibiotic resistant bacterial infections. Due to the high host specificity of bacteriophages, which can be strain-specific, and to thwart development of bacterial resistance, phage cocktails of 3–10 different bacteriophages are often used. However, combining multiple bacteriophages can have unintended side effects such as antagonistic interactions amongst component phages due to coinfection in the same host cell and competition for limited resources, or phage-phage competition for the same receptor site.^99^ In this study we examined the change in enteric phage-phage correlation networks over the first year of life and found sparse correlations at all timepoints in infants who later developed atopic disease, suggesting that phage-phage interactions, whether neutral, synergistic, or antagonistic, may be an important factor contributing to atopic disease development. These data provide insight into cooperative and antagonistic bacteriophage networks and potential markers for host bacterial communities within this cohort. While these data need to be validated in other cohorts, they suggest that therapies to restore normal phage-phage interaction networks in early infancy may affect risk of later atopic disease development. Further deep sequencing to examine genomic content of phages and identify possible interaction mechanisms in this cohort is currently underway.

One question this study leaves unanswered is the source of these enteric viruses, though we see hints of possible origins. While the infant gut bacteriome is thought to be partly seeded by the maternal gut bacteriome,^18^^,^^77^ limited data shows that maternal transmission of gut virome components is low (15%).^77^ This suggests that other environmental sources such as human milk^58^ may be more influential on virome acquisition and development. In our cohort, ROC significantly differed from OOM in the number of infants exclusively breastfed versus mixed human milk/formula fed, which may have impacted phageome composition between these groups. Our prior study also showed that human milk from OOM mothers were enriched in *Actinomyceta*, while *Lactobacillacae* were increased in ROC milk.^100^ This would fit with our finding of increased *Enterococcus* phages in 6-week-old ROC infant stools, the reported host of whom is a member of the *Lactobacillacae,* and *Mycobacterium* phages (bacterial host a member of *Actinomyceta*) in OOM. Further, these data support that human milk may play a central role in infant enteric phage acquisition, also suggested from other studies.^58^

Limitations to this study include low numbers of infants who developed an atopic disease by 2 y of age. Atopic outcomes in this whole cohort are only known for up to 2 y of age; however, allergic rhinitis and asthma usually develops after this time period. Follow-up on this cohort is ongoing, which may increase power for future studies by including outcomes of respiratory allergies. Fewer samples underwent virome analysis at 6 and 12 months, which likely impacts power to detect differences and feasibility of multivariate analysis at these timepoints. Genetic differences between lifestyle groups may contribute to different rates of atopic diseases between groups, although the dramatic increase in atopic disease in just a few decades suggests a role for environmental factors. Limitations to viromic detection tools include inaccurate identification of viral sequences, such as due to sparse viral reads and database biases. Finally, the RNA virome was not assessed, and could further contribute to understanding the role of the enteric virome in atopic disease development. Nonetheless, further analysis of eukaryotic viruses using higher sequencing depth or methods to enrich for viral sequencing is supported by these findings. A better understanding of how the early life enteric virome impacts development of atopic diseases could provide early biomarkers to high-risk infants allowing therapeutics or preventative measures to abrogate onset of atopic disease.

From these results it is clear that studying all components of the enteric microbiome beyond just bacteria is vital to understanding how the infant enteric microbiome contributes to atopic disease development. Pathogenesis of atopic disease is complex, including genetic and environmental factors. These data suggest a model in which *B. infantis* acts as a lodestone species within the infant gut microbiome, attracting beneficial bacterial and viral communities and repelling higher inflammatory microbial communities. Early development of sparse phage-phage interactions and bacterial-phage networks lacking *B. infantis* promotes immune dysregulation and possibly atopic disease development in at-risk infants. Future studies expanding these current findings to earlier timepoints and host response to enteric viruses are needed before a full understanding of the influence the virome exerts on atopic disease development can be established.

## Supplementary Material

Video1.docx Video1.docx

Supplemental Figures and Tables_revised.pdf Supplemental Figures and Tables_revised.pdf

## Data Availability

Metagenomic sequencing data is available at BioProject ID PRJNA1290379 (http://www.ncbi.nlm.nih.gov/bioproject/1290379).

## Abbreviations

AD: atopic dermatitis
CI: confidence interval
FA: food allergy
DA: differentially abundant
OOM: old order mennonite
ROC: urban/suburban Rochester, NY infant populations
ZOOM: “Zooming in on Old Order Mennonites” cohort
