# Supplementary material for: The enteric DNA virome differs in infants at risk for atopic disease
Source: Gut Microbes. 2026 Jan 27;18(1):2616066. doi: 10.1080/19490976.2026.2616066 (PMC12851394; doi:10.1080/19490976.2026.2616066)
Supplement: Video1.docx [file KGMI_A_2616066_SM0827.docx]

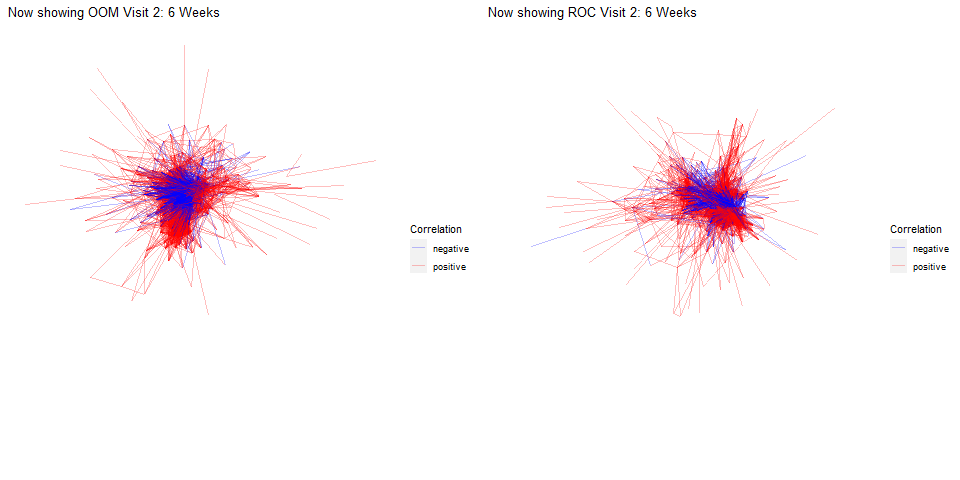


**A**

**B**

**C**


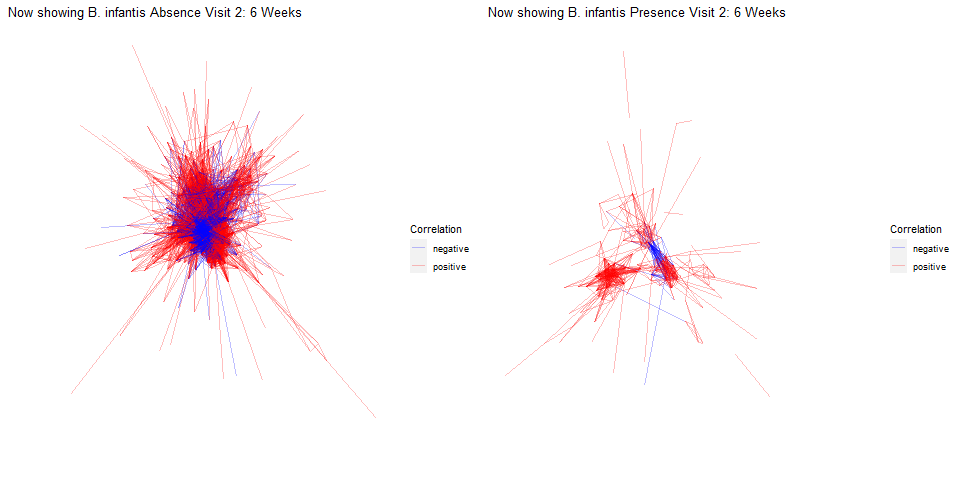


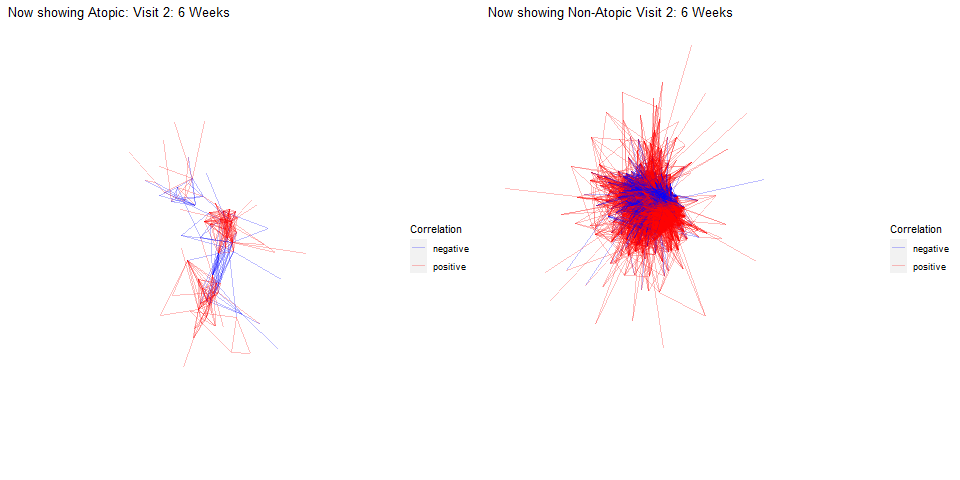


**Video1: Bacteriophage-bacteriophage associations within infant groups over the first year of life.** Significant associations (*p_adj_*<0.01) between bacteriophage species present in at least 25% of infants by (A) OOM vs ROC, (B) *B. infantis* absence/presence, and (C) atopic disease status by 2 years of age are mapped and animated at 6 weeks, 6 months, and 12 months. Red lines indicate positive phage-phage associations, blue lines indicate negative phage-phage association.
