# Supplementary material for: The enteric DNA virome differs in infants at risk for atopic disease
Source: Gut Microbes. 2026 Jan 27;18(1):2616066. doi: 10.1080/19490976.2026.2616066 (PMC12851394; doi:10.1080/19490976.2026.2616066)
Supplement: Supplemental Figures and Tables_revised.pdf [file KGMI_A_2616066_SM0826.pdf]

**Supplementary Information**

## The Enteric DNA Virome Differs in Infants at Risk for Atopic Disease

**Authors:** Tyrus J. Perdue[1,†], Cassandra E. Newkirk[2, †], Robert Beblavy[3], Antti E. Seppo[4], Erin C. Davis[4], Michael Sohn[3], Kirsi M. Järvinen[1, 4, 5,], Cynthia L. Monaco[1, 2, *]

[1] Department of Microbiology and Immunology, University of Rochester Medical Center, Rochester, NY, 14642, United States

[2] Department of Internal Medicine, Division of Infectious Diseases, University of Rochester Medical Center, Rochester, NY, 14642, United States

[3] Department of Biostatistics and Computational Biology, University of Rochester Medical Center, Rochester, NY, 14642, United States

[4] Division of Allergy and Immunology, Center for Food Allergy, Department of Pediatrics, University of Rochester School of Medicine and Dentistry, Golisano Children's Hospital, Rochester, NY, 14642, United States

[5] Division of Allergy, Immunology, and Rheumatology, Department of Medicine, University of Rochester School of Medicine and Dentistry, Rochester, NY, 14642, United States

[†] These authors contributed equally

* Corresponding Author. Email: Cynthia_Monaco@URMC.Rochester.edu

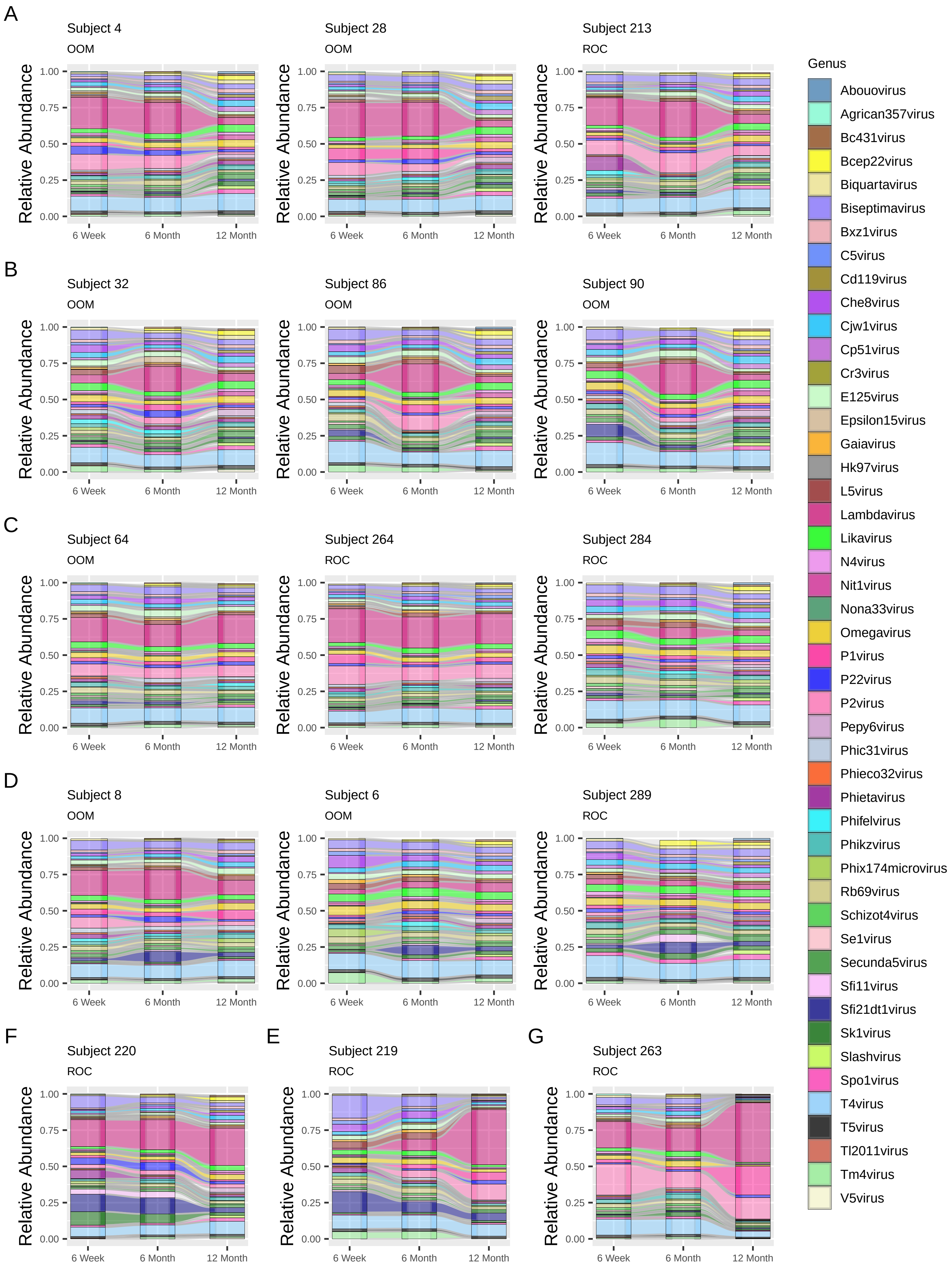
A
Subject 4
OOM
Subject 28
OOM
Subject 213
ROC
B
Subject 32
OOM
Subject 86
OOM
Subject 90
OOM
C
Subject 64
OOM
Subject 264
ROC
Subject 284
ROC
D
Subject 8
OOM
Subject 6
OOM
Subject 289
ROC
F
Subject 220
ROC
E
Subject 219
ROC
G
Subject 263
ROC
Relative Abundance
1.00
0.75
0.50
0.25
0.00
6 Week
6 Month
12 Month
Genus
Abouovirus
Agrican357virus
Bc431virus
Bcep22virus
Biquartavirus
Biseptimavirus
Bxz1virus
C5virus
Cd119virus
Che8virus
Cjw1virus
Cp51virus
Cr3virus
E125virus
Epsilon15virus
Gaiavirus
Hk97virus
L5virus
Lambdavirus
Likavirus
N4virus
Nit1virus
Nona33virus
Omegavirus
P1virus
P22virus
P2virus
Pepy6virus
Phic31virus
Phieco32virus
Phietavirus
Phifelvirus
Phikzvirus
Phix174microvirus
Rb69virus
Schizot4virus
Se1virus
Secunda5virus
Sfi11virus
Sfi21dt1virus
Sk1virus
Slashvirus
Spo1virus
T4virus
T5virus
Tl2011virus
Tm4virus
V5virus

**Figure S1: Alluvial diagram from subset of subjects showing shared bacteriophage relative abundance changes over time.** Alluvial diagrams were made for a subset of subjects analyzed at all timepoints and grouped by shared patterns. A) "bottle" pattern for lambdavirus (fuschia) changes over time, with decreased abundance at 12 months; B) expansion of lambdavirus at 6 months; C) Stable phage populations over all timepoints; D) expansion of Sfi21dt1 viruses (blue-purple) at 6 months; E) "reverse bottle" patter of lambdavirus with expansion of these phages at 12 months. Note that some subjects exhibit more than one pattern. For example, subject 8 also has "bottle" pattern for lambdavirus; subjects 219 and 220 exhibit a "bottle" pattern for Sfi21dt1 viruses as well.

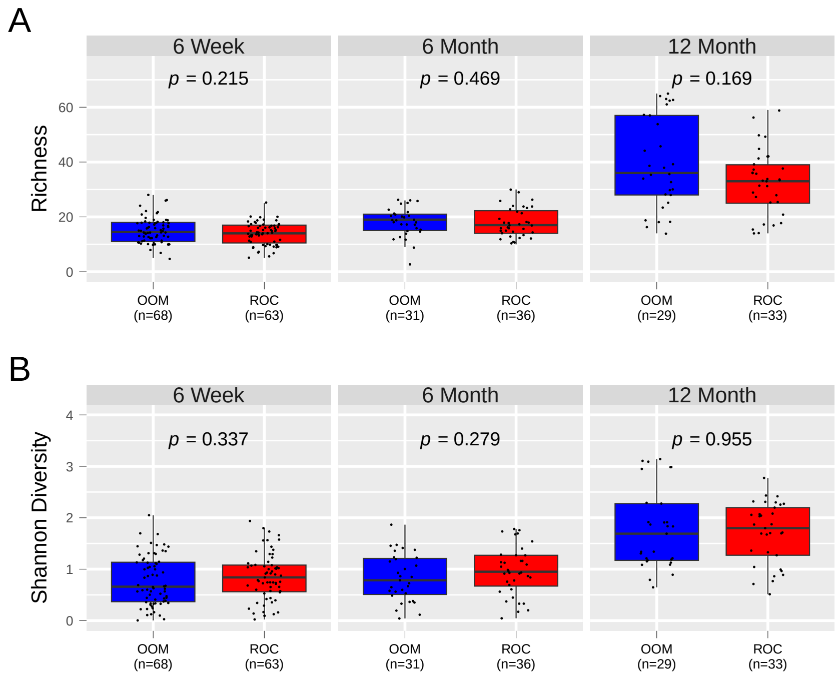


**Figure S2: Enteric bacterial species alpha diversity metrics between infants in the OOM and ROC group. (A)** Bacterial total species count and **(B)** Shannon diversity separated by lifestyle group across the 3 timepoints. OOM, blue. ROC, red.

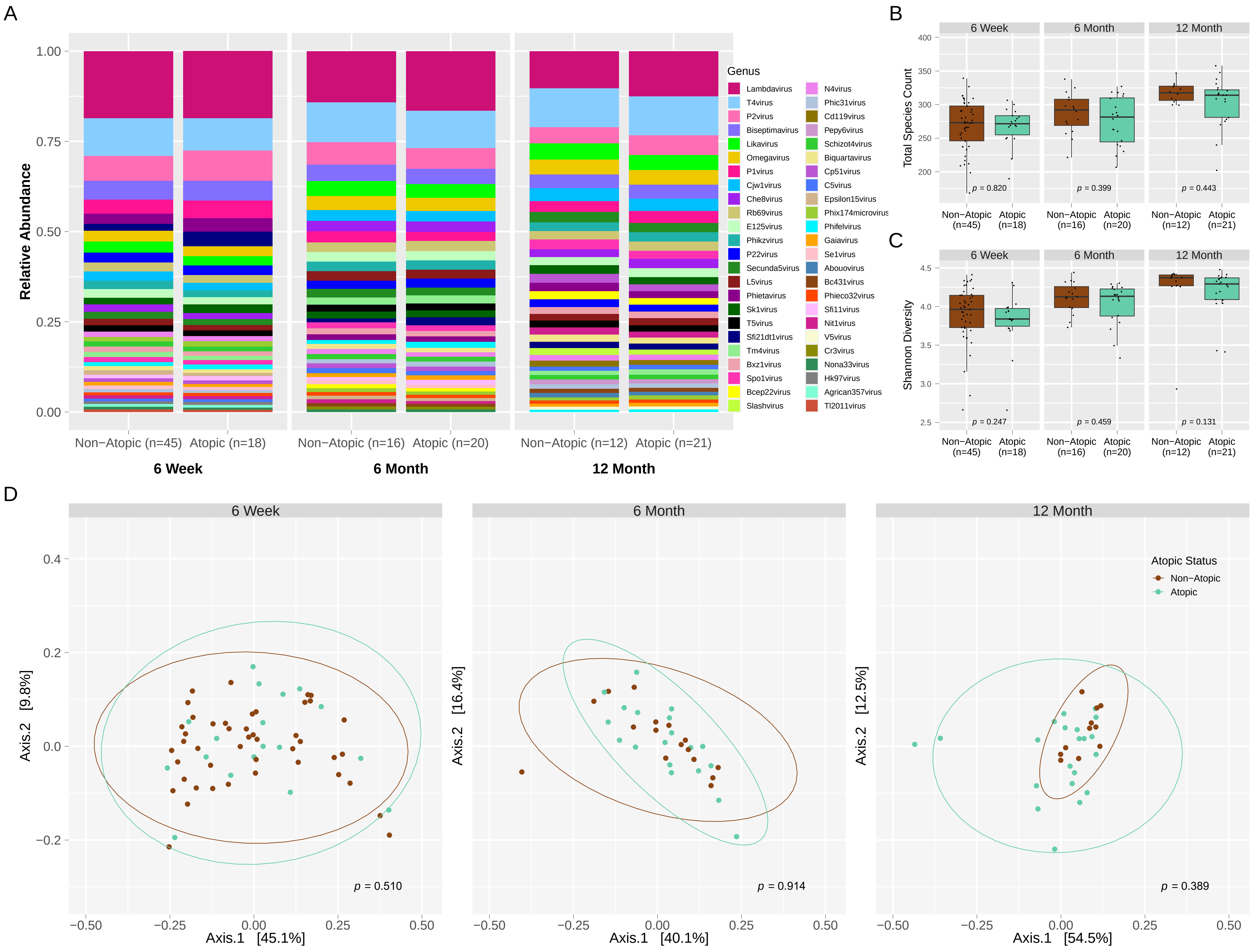

A
Relative Abundance
1.00
0.75
0.50
0.25
0.00
Non−Atopic (n=45)
Atopic (n=18)
Non−Atopic (n=16)
Atopic (n=20)
Non−Atopic (n=12)
Atopic (n=21)
6 Week
6 Month
12 Month
Genus
Lambdavirus
T4virus
P2virus
Biseptimavirus
Likavirus
Omegavirus
P1virus
Cjw1virus
Che8virus
Rb69virus
E125virus
Phikzvirus
P22virus
Secunda5virus
L5virus
Phietavirus
Sk1virus
T5virus
Sfi21dt1virus
Tm4virus
Bxz1virus
Spo1virus
Bcep22virus
Slashvirus
N4virus
Phic31virus
Cd119virus
Pepy6virus
Schizot4virus
Biquartavirus
Cp51virus
C5virus
Epsilon15virus
Phix174microvirus
Phifelvirus
Gaiavirus
Se1virus
Abouovirus
Bc431virus
Phieco32virus
Sfi11virus
Nit1virus
V5virus
Cr3virus
Nona33virus
Hk97virus
Agrican357virus
Tl2011virus
B
Total Species Count
6 Week
6 Month
12 Month
p = 0.820
p = 0.399
p = 0.443
C
Shannon Diversity
p = 0.247
p = 0.459
p = 0.131
D
Axis.2 [9.8%]
Axis.1 [45.1%]
p = 0.510
Axis.2 [16.4%]
Axis.1 [40.1%]
p = 0.914
Axis.2 [12.5%]
Axis.1 [54.5%]
p = 0.389
Atopic Status
Non−Atopic
Atopic

**Figure S3: Enteric bacteriophage populations between non-atopic and atopic infants in the ROC group.** **(A)** Relative abundance bar graphs comparing the 40 most abundant genera across the three timepoints by atopic status by 2 years of age. **(B)** Bacteriophage species count and **(C)** phage genera Shannon diversity separated by atopic status across the 3 timepoints. **(D)** Principle coordinate analysis of phage genera using the Bray-Curtis dissimilarity index comparing bacteriophage populations by atopic status across the three timepoints. Non-atopic = brown, atopic = aquamarine; 6 weeks: non-atopic, n = 45, atopic, n = 18; 6 months: non-atopic, n = 16, atopic, n = 20; 12 months: non-atopic, n = 12, atopic, n = 21.

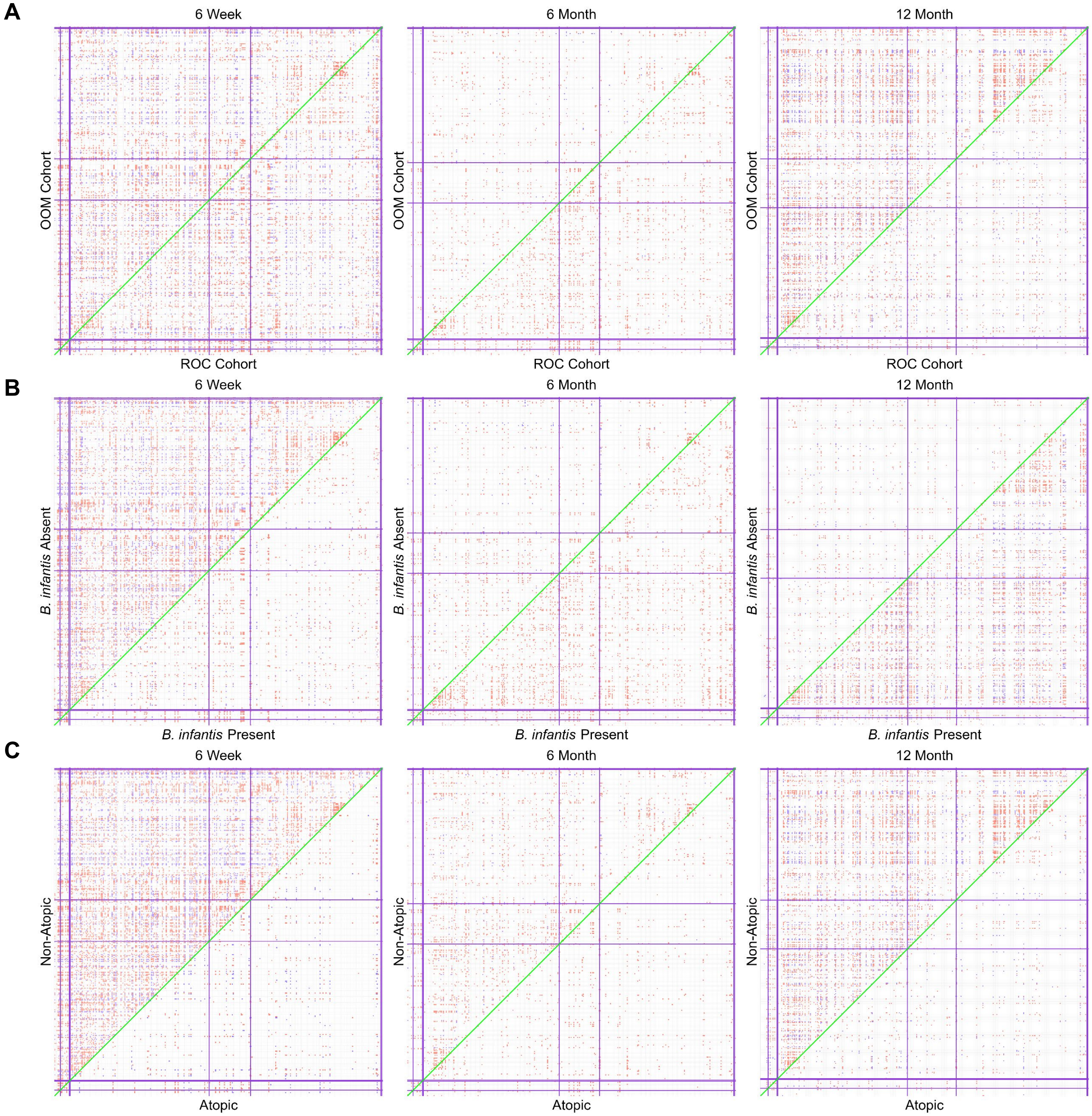


**Figure S4: Correlation plots analysis of significant associations between bacteriophage species across all three timepoints.** Correlations between bacteriophage species present in at least 25% of samples at each visit were determined by Kendall correlation coefficient with the Benjamini-Hochberg procedure multiple comparisons correction at each timepoint. Each line on the X and Y axis represents a different phage species. Significant correlations ($p_{adj}$<0.05) are shown as either red (positive correlations) or blue (negative correlation) dots. Non-significant correlations are uncolored. Phage families are grouped and separated by purple lines. **(A)** Bacteriophage associations within OOM (left upper triangle), ROC (right lower triangle); **(B)** *B. infantis* absent (left upper triangle), B. infantis present (right lower triangle); (C) Non-atopic infants (left upper triangle), atopic infants (right lower triangle).

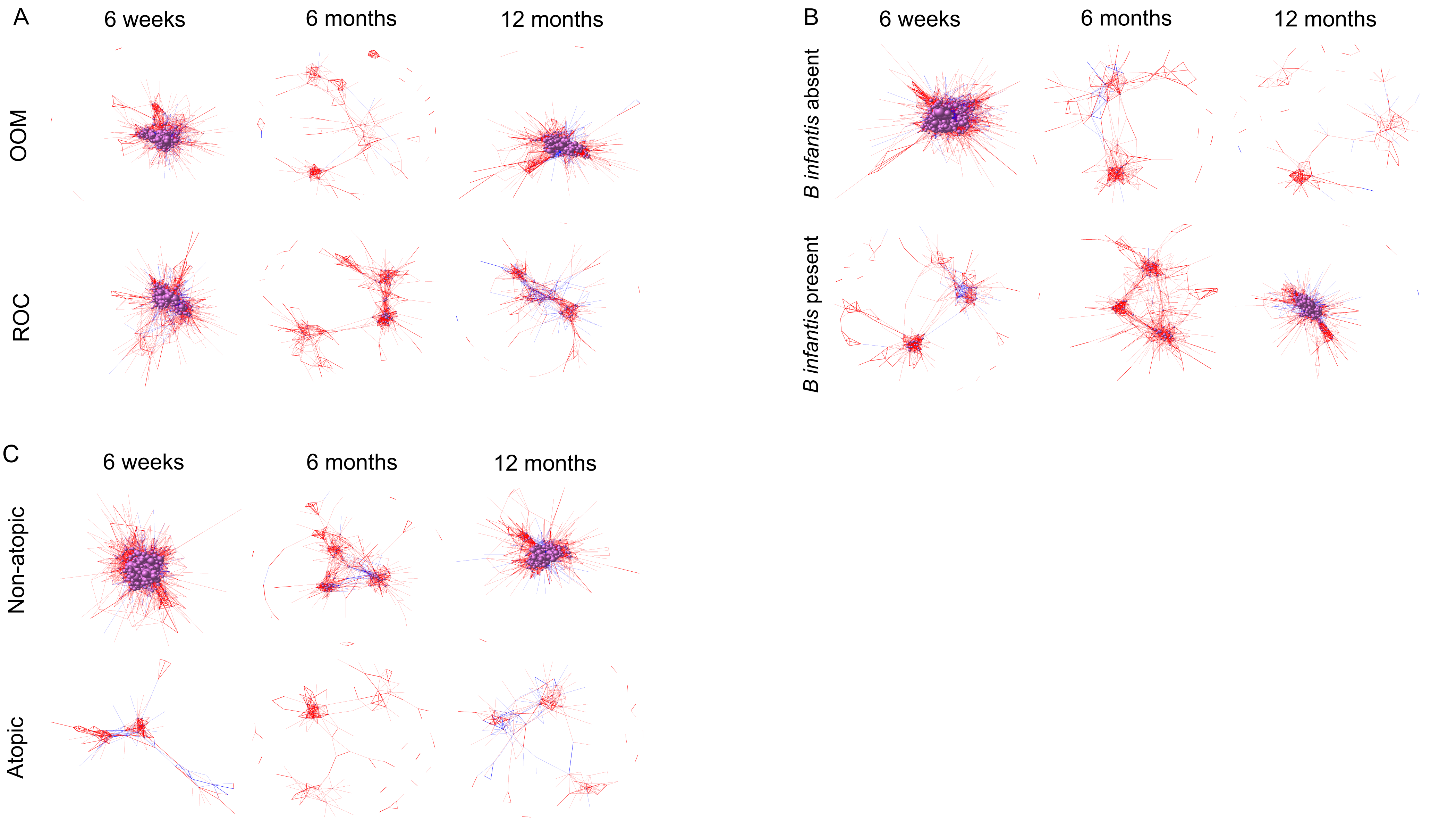


**Figure S5: Network analysis of enteric bacteriophage-bacteriophage associations within comparator groups across the first year of life.** Significant associations (*padj*<0.01) between bacteriophage species present in at least 25% of infants within the OOM group and ROC group (**A**), infants without and with enteric *B. infantis* colonization (**B**), and without or with atopic disease by 2 years of age (**C**) are mapped at 6 weeks, 6 months, and 12 months. Line thickness indicates strength of associations (i.e. thicker lines represent higher value correlation coefficient), red = positive association, blue = negative association. Purple nodes denote phage species, with larger sphere size indicating more numbers of significant associations to other species.

**Video 1: Bacteriophage-bacteriophage associations within infant groups over the first year of life.** Significant associations (padj<0.01) between bacteriophage species present in at least 25% of infants by OOM vs ROC, B. infantis absence/presence, and atopic disease status, respectively, by 2 years of age are mapped and animated at 6 weeks, 6 months, and 12 months. Red lines indicate positive phage-phage associations, blue lines indicate negative phage-phage association.

**Table S1: Number of participants who developed the indicated atopic disease by 2 years of age**

| | OOM | ROC |
|---|---|---|
| **Atopic dermatitis** | 2 | 23 |
| **IgE-mediated food allergy** | 0 | 9 |
| **Allergic proctocolitis** | 0 | 2 |

**Table S2: Differentially abundant enteric phage taxa between ROC (red) and OOM (blue) infants using a linear mixed effects model.**

| Species | base Mean | $log_2$-Fold Change | lfcSE | stat | pvalue | padj | Visit Tested |
|---|---|---|---|---|---|---|---|
| Mycobacterium virus Tweety | 827.53 | -1.3816 | 0.3669 | -3.7658 | 0.0003 | 0.02174 | 6 Week |
| Mycobacterium virus Godines | 283.61 | -1.1675 | 0.3439 | -3.3950 | 0.0009 | 0.02709 | 6 Week |
| Mycobacterium virus Rey | 3117.60 | -1.0513 | 0.2967 | -3.5437 | 0.0006 | 0.02709 | 6 Week |
| Streptococcus virus Cp1 | 3950.08 | -1.0431 | 0.3467 | -3.0087 | 0.0032 | 0.04804 | 6 Week |
| Pseudomonas virus PaMx28 | 1043.85 | -0.9298 | 0.2481 | -3.7481 | 0.0003 | 0.02174 | 6 Week |
| Mycobacterium virus Giles | 3948.85 | -0.8566 | 0.2697 | -3.1764 | 0.0019 | 0.03261 | 6 Week |
| Cellulophaga virus Cba41 | 831.46 | -0.7982 | 0.2540 | -3.1427 | 0.0021 | 0.03373 | 6 Week |
| Mycobacterium virus Barnyard | 1313.59 | -0.7979 | 0.2309 | -3.4563 | 0.0007 | 0.02709 | 6 Week |
| Salmonella virus FSLSP030 | 101.06 | -0.6689 | 0.1534 | -4.3601 | 0.0000 | 0.00589 | 6 Week |
| Halobacterium virus phiH | 122.31 | -0.6662 | 0.2082 | -3.2003 | 0.0017 | 0.03261 | 6 Week |
| Synechococcus phage S-RIM8 | 6465.36 | -0.6408 | 0.1691 | -3.7897 | 0.0002 | 0.02174 | 6 Week |
| Mycobacterium virus Sirduracell | 2575.70 | -0.6382 | 0.1904 | -3.3511 | 0.0011 | 0.02709 | 6 Week |
| Escherichia virus FV3 | 2601.39 | -0.6130 | 0.1917 | -3.1980 | 0.0017 | 0.03261 | 6 Week |
| Escherichia virus E112 | 1626.98 | -0.6018 | 0.1724 | -3.4899 | 0.0007 | 0.02709 | 6 Week |
| Synechococcus phage ACG-2014c | 6270.31 | -0.5535 | 0.1598 | -3.4635 | 0.0007 | 0.02709 | 6 Week |
| Mycobacterium virus Wildcat | 10838.63 | -0.5214 | 0.1495 | -3.4884 | 0.0007 | 0.02709 | 6 Week |
| Mycobacterium virus Chrisnmich | 82.62 | -0.3800 | 0.1193 | -3.1847 | 0.0018 | 0.03261 | 6 Week |
| Streptomyces virus Zemlya | 30628.80 | -0.3763 | 0.1124 | -3.3479 | 0.0011 | 0.02709 | 6 Week |
| Pseudomonas virus PaMx25 | 9838.17 | -0.3611 | 0.1202 | -3.0047 | 0.0032 | 0.04804 | 6 Week |
| Mycobacterium virus Thibault | 18839.54 | -0.3559 | 0.0975 | -3.6504 | 0.0004 | 0.02566 | 6 Week |
| Salmonella virus LSPA1 | 89.34 | 0.6226 | 0.1835 | 3.3932 | 0.0009 | 0.02709 | 6 Week |
| Erwinia virus Ea35-70 | 10196.76 | 0.6294 | 0.1904 | 3.3050 | 0.0012 | 0.02775 | 6 Week |
| Bacillus virus Pony | 3079.65 | 0.6338 | 0.1462 | 4.3357 | 0.0000 | 0.00589 | 6 Week |
| Shigella virus Shfl2 | 1554.51 | 0.6850 | 0.2023 | 3.3854 | 0.0009 | 0.02709 | 6 Week |
| Enterococcus virus FL3 | 3824.90 | 0.9237 | 0.2918 | 3.1662 | 0.0019 | 0.03261 | 6 Week |
| Enterococcus virus FL1 | 1625.39 | 0.9958 | 0.3004 | 3.3154 | 0.0012 | 0.02775 | 6 Week |
| Escherichia virus M13 | 577.23 | 1.1105 | 0.3495 | 3.1774 | 0.0019 | 0.03261 | 6 Week |
| Mycobacterium virus Konstantine | 203.21 | -2.0797 | 0.3952 | -5.2622 | 0.0000 | 0.00062 | 6 Month |
| Escherichia virus CVM10 | 4440.16 | -2.0315 | 0.4973 | -4.0851 | 0.0001 | 0.00635 | 6 Month |
| Bacillus virus B103 | 636.88 | -1.9860 | 0.5667 | -3.5044 | 0.0008 | 0.02145 | 6 Month |
| Pseudomonas virus PaMx28 | 1550.12 | -1.5590 | 0.3048 | -5.1143 | 0.0000 | 0.00062 | 6 Month |
| Mycobacterium virus Bongo | 803.64 | -1.4532 | 0.4464 | -3.2552 | 0.0018 | 0.03807 | 6 Month |
| Escherichia virus If1 | 7379.79 | -1.3983 | 0.4023 | -3.4760 | 0.0009 | 0.02145 | 6 Month |
| Escherichia virus JSE | 403.63 | -1.3978 | 0.3164 | -4.4172 | 0.0000 | 0.00399 | 6 Month |
| Mycobacterium virus Godines | 911.46 | -1.2174 | 0.3962 | -3.0724 | 0.0031 | 0.04527 | 6 Month |
| Streptococcus virus SPQS1 | 257.43 | -1.1037 | 0.3403 | -3.2431 | 0.0019 | 0.03807 | 6 Month |
| Escherichia virus AR1 | 1155.70 | -0.9011 | 0.2975 | -3.0292 | 0.0035 | 0.04527 | 6 Month |
| Escherichia virus E112 | 2060.33 | -0.6449 | 0.1748 | -3.6888 | 0.0005 | 0.01587 | 6 Month |
| Mycobacterium virus Sebata | 3931.14 | -0.6289 | 0.2030 | -3.0976 | 0.0029 | 0.04527 | 6 Month |
| Synechococcus phage S-RIM2 | 2550.51 | -0.5313 | 0.1503 | -3.5361 | 0.0008 | 0.02072 | 6 Month |
| Enterococcus virus phiEC24C | 48798.71 | -0.4368 | 0.1260 | -3.4673 | 0.0009 | 0.02145 | 6 Month |
| Bacillus virus CP51 | 9479.95 | 0.3119 | 0.1013 | 3.0795 | 0.0030 | 0.04527 | 6 Month |
| Vibrio phage phi16 | 4605.42 | 0.3365 | 0.1050 | 3.2043 | 0.0021 | 0.03832 | 6 Month |
| Escherichia virus V5 | 1568.70 | 0.5057 | 0.1340 | 3.7729 | 0.0004 | 0.01447 | 6 Month |
| Escherichia virus AHS24 | 19.36 | 0.6583 | 0.2167 | 3.0375 | 0.0034 | 0.04527 | 6 Month |
| Staphylococcus virus SEP9 | 1123.89 | 0.7923 | 0.2163 | 3.6637 | 0.0005 | 0.01589 | 6 Month |
| Escherichia virus ECML4 | 81.79 | 0.8083 | 0.2531 | 3.1934 | 0.0022 | 0.03832 | 6 Month |
| Klebsiella virus KP27 | 924.84 | 0.8475 | 0.2788 | 3.0397 | 0.0034 | 0.04527 | 6 Month |
| Propionibacterium virus PAS50 | 90.03 | 0.8752 | 0.2867 | 3.0532 | 0.0033 | 0.04527 | 6 Month |
| Bacillus virus Pony | 1828.73 | 1.0004 | 0.2338 | 4.2790 | 0.0001 | 0.00519 | 6 Month |
| Roseobacter virus RDJL1 | 766.49 | 1.1229 | 0.2310 | 4.8615 | 0.0000 | 0.00106 | 6 Month |
| Streptomyces virus R4 | 112.04 | 1.1413 | 0.3585 | 3.1837 | 0.0022 | 0.03832 | 6 Month |
| Acinetobacter virus AB2 | 107.71 | 1.1596 | 0.3752 | 3.0909 | 0.0029 | 0.04527 | 6 Month |
| Mycobacterium virus Mosmoris | 103.29 | 1.2988 | 0.3076 | 4.2219 | 0.0001 | 0.00528 | 6 Month |
| Mycobacterium virus Twister | 250.83 | 1.3014 | 0.3524 | 3.6935 | 0.0005 | 0.01587 | 6 Month |

| | | | | | | | |
|---|---|---|---|---|---|---|---|
| Bacillus virus Slash | 1742.43 | 1.3042 | 0.3322 | 3.9257 | 0.0002 | 0.00968 | 6 Month |
| Listeria phage LMSP-25 | 874.14 | 1.3430 | 0.3236 | 4.1497 | 0.0001 | 0.00581 | 6 Month |
| Bacillus virus Staley | 1243.41 | 1.5023 | 0.4176 | 3.5978 | 0.0006 | 0.01824 | 6 Month |
| Escherichia virus 191 | 553.18 | 1.7247 | 0.5339 | 3.2305 | 0.0019 | 0.03807 | 6 Month |
| Mycobacterium virus Godines | 542.40 | -1.6384 | 0.4038 | -4.0579 | 0.0001 | 0.02049 | 12 Month |
| Bacillus virus Bobb | 6062.22 | 0.4163 | 0.0814 | 5.1175 | 0.0000 | 0.00145 | 12 Month |
| Bacillus virus Hakuna | 739.95 | 0.6210 | 0.1631 | 3.8082 | 0.0003 | 0.02804 | 12 Month |
| Staphylococcus virus SEP9 | 1608.16 | 0.8324 | 0.1973 | 4.2190 | 0.0001 | 0.01781 | 12 Month |
| Bacillus virus SPG24 | 159.01 | 1.3338 | 0.3491 | 3.8210 | 0.0003 | 0.02804 | 12 Month |
| Escherichia virus M13 | 141.00 | 1.6847 | 0.4581 | 3.6775 | 0.0005 | 0.03561 | 12 Month |

**Table S3: Differentially abundant enteric bacterial species between ROC (red) and OOM (blue) infants using a linear mixed effects model.**

| | base Mean | log2-Fold Change | lfcSE | stat | pvalue | padj | Age Tested |
|---|---|---|---|---|---|---|---|
| Streptococcus_oralis | 433.83 | 0.6692 | 0.1935 | 3.4584 | 0.0007 | 0.00848 | 6 weeks |
| Streptococcus_thermophilus | 891.39 | 0.9967 | 0.2785 | 3.5784 | 0.0005 | 0.00848 | 6 weeks |
| Flavonifractor_plautii | 2801.19 | 1.1139 | 0.3726 | 2.9895 | 0.0033 | 0.02582 | 6 weeks |
| Klebsiella_michiganensis | 1462.02 | 1.3008 | 0.3330 | 3.9063 | 0.0002 | 0.00596 | 6 weeks |
| Haemophilus_parainfluenzae | 228.20 | 1.3964 | 0.4060 | 3.4395 | 0.0008 | 0.00848 | 6 weeks |
| Enterobacter_cloacae_complex | 1526.83 | 1.7229 | 0.4532 | 3.8015 | 0.0002 | 0.00596 | 6 weeks |
| Enterococcus_faecalis | 497.48 | 3.2692 | 0.9707 | 3.3679 | 0.0010 | 0.00899 | 6 weeks |
| Actinomyces_sp_oral_taxon_181 | 84.79 | -3.2851 | 1.0311 | -3.1862 | 0.0022 | 0.01931 | 6 months |
| Streptococcus_mitis | 1142.32 | -3.1710 | 0.6126 | -5.1766 | 0.0000 | 0.00007 | 6 months |
| Actinomyces_odontolyticus | 56.99 | -2.4549 | 0.6685 | -3.6726 | 0.0005 | 0.00743 | 6 months |
| Streptococcus_peroris | 85.87 | -2.3533 | 0.6579 | -3.5769 | 0.0007 | 0.00809 | 6 months |
| Streptococcus_pseudopneumoniae | 63.58 | -1.5520 | 0.2710 | -5.7269 | 0.0000 | 0.00002 | 6 months |
| Bifidobacterium_longum | 921711.73 | -1.4956 | 0.5023 | -2.9774 | 0.0041 | 0.03096 | 6 months |
| Enterococcus_avium | 1418.20 | -1.4898 | 0.5071 | -2.9379 | 0.0046 | 0.03096 | 6 months |
| Eggerthella_lenta | 1059.89 | 2.4082 | 0.6412 | 3.7559 | 0.0004 | 0.00743 | 6 months |
| Ruminococcus_gnavus | 71.19 | 3.4520 | 1.0774 | 3.2041 | 0.0021 | 0.01931 | 6 months |
| Collinsella_aerofaciens | 5198.60 | -4.3367 | 1.3438 | -3.2270 | 0.0020 | 0.03005 | 12 months |
| Blautia_obeum | 492.41 | -3.1514 | 1.0068 | -3.1302 | 0.0027 | 0.03005 | 12 months |
| Eubacterium_hallii | 1553.76 | -2.6147 | 0.8547 | -3.0591 | 0.0033 | 0.03086 | 12 months |
| Dorea_longicatena | 5417.17 | -2.1211 | 0.5413 | -3.9186 | 0.0002 | 0.00699 | 12 months |
| Blautia_sp | 120.28 | -1.9541 | 0.5402 | -3.6174 | 0.0006 | 0.01233 | 12 months |
| Agathobaculum_butyriciproducens | 1392.27 | -1.6534 | 0.5340 | -3.0960 | 0.0030 | 0.03005 | 12 months |
| Firmicutes_bacterium_CAG_56 | 1027.60 | -1.0209 | 0.3675 | -2.7779 | 0.0073 | 0.04644 | 12 months |
| Dorea_formicigenerans | 10579.39 | -0.7938 | 0.2750 | -2.8863 | 0.0054 | 0.03851 | 12 months |
| Streptococcus_lutetiensis | 211.75 | 1.3307 | 0.4675 | 2.8461 | 0.0060 | 0.04066 | 12 months |
| Bacteroides_ovatus | 642.34 | 1.5894 | 0.5294 | 3.0022 | 0.0039 | 0.03149 | 12 months |
| Clostridium_bolteae | 130.84 | 1.7641 | 0.5840 | 3.0207 | 0.0037 | 0.03149 | 12 months |
| Eggerthella_lenta | 13432.68 | 2.3946 | 0.8260 | 2.8991 | 0.0052 | 0.03851 | 12 months |
| Veillonella_sp_CAG_933 | 263.63 | 2.4163 | 0.5321 | 4.5408 | 0.0000 | 0.00167 | 12 months |
| Hungatella_hathewayi | 364.22 | 2.4852 | 0.5427 | 4.5789 | 0.0000 | 0.00167 | 12 months |
| Anaerostipes_caccae | 123.15 | 2.6598 | 0.7243 | 3.6723 | 0.0005 | 0.01233 | 12 months |
| Tyzzerella_nexilis | 106.25 | 2.7075 | 0.6409 | 4.2247 | 0.0001 | 0.00333 | 12 months |
| Erysipelatoclostridium_ramosum | 286.75 | 2.7930 | 0.8807 | 3.1713 | 0.0024 | 0.03005 | 12 months |
| Ruminococcus_gnavus | 7297.50 | 3.0437 | 0.9781 | 3.1117 | 0.0028 | 0.03005 | 12 months |
| Streptococcus_salivarius | 471.48 | 3.4139 | 1.0616 | 3.2158 | 0.0021 | 0.03005 | 12 months |

**Table S4: Differentially abundant enteric phage species in infants with *B infantis* (blue) colonization compared to those without colonization (orange) using a linear mixed effects model, accounting for lifestyle.**

| Species | Base Mean | $log_2$-fold Change | lfcSE | stat | pvalue | padj | Age Tested |
|---|---|---|---|---|---|---|---|
| Pseudomonas virus F8 | 410.8792 | -1.18341 | 0.270064 | -4.38197 | 2.43E-05 | 0.001551 | 6 weeks |
| Edwardsiella virus PEi21 | 186.9968 | -1.18292 | 0.296156 | -3.99424 | 0.000109 | 0.004909 | 6 weeks |
| Staphylococcus virus SEP9 | 1936.06 | -1.01136 | 0.278267 | -3.63451 | 0.000402 | 0.010657 | 6 weeks |
| Brucella virus Tb | 341.1699 | -1.01009 | 0.315336 | -3.20321 | 0.001715 | 0.027854 | 6 weeks |
| Bacillus virus Slash | 3534.844 | -1.00708 | 0.321029 | -3.13702 | 0.002118 | 0.030713 | 6 weeks |
| Klebsiella virus KP32 | 201.4713 | -1.00071 | 0.343003 | -2.91749 | 0.004169 | 0.041692 | 6 weeks |
| Shigella virus AG3 | 339.3671 | -0.94802 | 0.331921 | -2.85614 | 0.005005 | 0.047432 | 6 weeks |
| Xanthomonas virus OP2 | 1440.074 | -0.92604 | 0.313372 | -2.95507 | 0.003722 | 0.039775 | 6 weeks |
| Listeria phage LMSP-25 | 1824.348 | -0.91268 | 0.319685 | -2.85492 | 0.005024 | 0.047432 | 6 weeks |
| Shigella virus Shfl2 | 2178.503 | -0.86405 | 0.232817 | -3.71129 | 0.000306 | 0.009666 | 6 weeks |
| Pseudomonas virus PA7 | 2697.043 | -0.86381 | 0.26095 | -3.31023 | 0.001211 | 0.024589 | 6 weeks |
| Bacillus virus Pony | 4106.008 | -0.72362 | 0.164809 | -4.39066 | 2.34E-05 | 0.001551 | 6 weeks |
| Vibrio virus VHML | 2849.265 | -0.59695 | 0.191657 | -3.11467 | 0.002273 | 0.030761 | 6 weeks |
| Escherichia virus V5 | 2398.826 | -0.4613 | 0.14994 | -3.07655 | 0.002561 | 0.032499 | 6 weeks |
| Bacillus virus JL | 5936.523 | -0.35046 | 0.123572 | -2.83607 | 0.005311 | 0.047916 | 6 weeks |
| Escherichia virus CC31 | 3263.221 | 0.372713 | 0.122926 | 3.032011 | 0.002941 | 0.035122 | 6 weeks |
| Streptomyces virus Zemlya | 27593.99 | 0.376 | 0.132493 | 2.837873 | 0.005283 | 0.047916 | 6 weeks |
| Pseudomonas virus EL | 9454.46 | 0.3876 | 0.125542 | 3.087407 | 0.002476 | 0.032428 | 6 weeks |
| Mycobacterium virus Thibault | 16841.77 | 0.397672 | 0.113236 | 3.511885 | 0.000615 | 0.013878 | 6 weeks |
| Pseudomonas virus PaMx25 | 8784.634 | 0.40069 | 0.141678 | 2.828177 | 0.005436 | 0.047974 | 6 weeks |
| Enterococcus virus phiEC24C | 37671.35 | 0.460142 | 0.129935 | 3.541322 | 0.000556 | 0.013279 | 6 weeks |
| Escherichia virus 4MG | 5611.759 | 0.523123 | 0.177529 | 2.946682 | 0.003818 | 0.039775 | 6 weeks |
| Mycobacterium virus Wildcat | 9222.574 | 0.535619 | 0.175293 | 3.055562 | 0.002734 | 0.033641 | 6 weeks |
| Escherichia virus Min27 | 5370.743 | 0.596845 | 0.185872 | 3.211059 | 0.001672 | 0.027854 | 6 weeks |
| Cronobacter virus CR8 | 6541.402 | 0.645592 | 0.221533 | 2.914204 | 0.00421 | 0.041692 | 6 weeks |
| Synechococcus phage syn9 | 5293.59 | 0.663361 | 0.168643 | 3.933516 | 0.000136 | 0.005541 | 6 weeks |
| Mycobacterium virus Sirduracell | 2054.047 | 0.718605 | 0.222291 | 3.232717 | 0.00156 | 0.027854 | 6 weeks |
| Halobacterium virus phiH | 95.71845 | 0.771789 | 0.243298 | 3.172201 | 0.001894 | 0.029578 | 6 weeks |
| Listeria virus LP302 | 96.05656 | 0.790577 | 0.24621 | 3.210985 | 0.001673 | 0.027854 | 6 weeks |
| Mycobacterium virus Bernal13 | 4383.219 | 0.873139 | 0.277521 | 3.146206 | 0.002057 | 0.030713 | 6 weeks |
| Mycobacterium virus Giles | 2864.777 | 0.983289 | 0.315609 | 3.115527 | 0.002267 | 0.030761 | 6 weeks |
| Mycobacterium virus Sebata | 2008.3 | 1.061149 | 0.211868 | 5.008538 | 1.78E-06 | 0.000241 | 6 weeks |
| Escherichia virus AR1 | 508.7638 | 1.070263 | 0.315921 | 3.387759 | 0.000937 | 0.020016 | 6 weeks |
| Mycobacterium virus Bongo | 257.3449 | 1.097755 | 0.36737 | 2.988141 | 0.003366 | 0.039041 | 6 weeks |
| Mycobacterium virus Violet | 89.74444 | 1.108091 | 0.376081 | 2.946418 | 0.003821 | 0.039775 | 6 weeks |
| Shigella virus UTAM | 1043.85 | 1.18082 | 0.231581 | 5.098946 | 1.20E-06 | 0.000241 | 6 weeks |
| Mycobacterium virus Tiger | 105.8578 | 1.183421 | 0.32673 | 3.622013 | 0.00042 | 0.010657 | 6 weeks |
| Mycobacterium virus Shilan | 299.7909 | 1.273657 | 0.431771 | 2.949841 | 0.003781 | 0.039775 | 6 weeks |
| Propionibacterium virus PHL114L00 | 197.9129 | 1.346129 | 0.369641 | 3.641718 | 0.000392 | 0.010657 | 6 weeks |
| Mycobacterium virus Reprobate | 121.272 | 1.364392 | 0.337082 | 4.047653 | 8.90E-05 | 0.004516 | 6 weeks |
| Mycobacterium virus Taj | 174.0904 | 1.382378 | 0.369777 | 3.738409 | 0.000278 | 0.009666 | 6 weeks |
| Mycobacterium virus Trixie | 251.3115 | 1.426761 | 0.439933 | 3.243132 | 0.001508 | 0.027854 | 6 weeks |
| Mycobacterium virus Rey | 1904.457 | 1.464255 | 0.336033 | 4.357479 | 2.67E-05 | 0.001551 | 6 weeks |
| Mycobacterium virus Faith1 | 566.0457 | 1.673972 | 0.451401 | 3.708394 | 0.00031 | 0.009666 | 6 weeks |
| Mycobacterium virus TM4 | 729.044 | 1.829353 | 0.380451 | 4.808385 | 4.20E-06 | 0.000426 | 6 weeks |
| Mycobacterium virus Godines | 136.0807 | 2.142306 | 0.3722 | 5.755801 | 6.04E-08 | 2.45E-05 | 6 weeks |
| Brevibacillus virus Abouo | 2931.864 | -1.25265 | 0.287256 | -4.36076 | 4.89E-05 | 0.00403 | 6 months |
| Bacillus virus Pony | 3083.698 | -0.89942 | 0.257277 | -3.49591 | 0.000871 | 0.036957 | 6 months |
| Yersinia virus R1RT | 2834.224 | 0.685039 | 0.174876 | 3.917292 | 0.000223 | 0.015312 | 6 months |
| Synechococcus phage Syn19 | 1017.67 | 0.830148 | 0.22573 | 3.677622 | 0.000489 | 0.028783 | 6 months |
| Mycobacterium virus Sebata | 2155.66 | 1.0087 | 0.218333 | 4.620011 | 1.95E-05 | 0.002674 | 6 months |
| Mycobacterium virus Solon | 38.95083 | 1.066353 | 0.305843 | 3.486601 | 0.000897 | 0.036957 | 6 months |
| Mycobacterium virus Turbido | 406.389 | 1.394551 | 0.399871 | 3.487503 | 0.000894 | 0.036957 | 6 months |
| Escherichia virus AR1 | 484.0798 | 1.432231 | 0.320564 | 4.467853 | 3.35E-05 | 0.003453 | 6 months |
| Shigella virus UTAM | 676.8531 | 1.907412 | 0.318851 | 5.982144 | 1.14E-07 | 2.35E-05 | 6 months |
| Mycobacterium virus Godines | 219.5418 | 2.400838 | 0.384663 | 6.241411 | 4.11E-08 | 1.69E-05 | 6 months |
| Mycobacterium virus Thibault | 18478.84 | 0.336047 | 0.112281 | 2.992899 | 0.004031 | 0.036279 | 12 months |
| Streptomyces virus Zemlya | 30613.83 | 0.356725 | 0.119097 | 2.99524 | 0.004004 | 0.036279 | 12 months |
| Bacillus virus Bc431 | 6672.404 | 0.36083 | 0.119542 | 3.018438 | 0.003748 | 0.036036 | 12 months |
| Bacillus virus Agate | 3573.102 | 0.384886 | 0.129372 | 2.975026 | 0.00424 | 0.036607 | 12 months |
| Vibrio virus KVP40 | 6516.319 | 0.387208 | 0.133928 | 2.891171 | 0.005365 | 0.043642 | 12 months |
| Pseudomonas virus MP1412 | 3201.714 | 0.402596 | 0.134068 | 3.002928 | 0.003918 | 0.036279 | 12 months |

| | | | | | | | |
|---|---|---|---|---|---|---|---|
| Mycobacterium virus Wildcat | 8416.083 | 0.447004 | 0.147198 | 3.036757 | 0.003557 | 0.034994 | 12 months |
| Vibrio virus SSP002 | 3100.976 | 0.469993 | 0.14364 | 3.272007 | 0.001787 | 0.022854 | 12 months |
| Bacillus virus Spock | 1844.144 | 0.485879 | 0.171627 | 2.831021 | 0.006335 | 0.049627 | 12 months |
| Aeromonas virus AS4 | 2652.748 | 0.512173 | 0.150724 | 3.398094 | 0.001221 | 0.017811 | 12 months |
| Vibrio virus nt1 | 5603.488 | 0.518449 | 0.157202 | 3.297976 | 0.001653 | 0.021855 | 12 months |
| Achromobacter phage JWAlpha | 5731.917 | 0.55396 | 0.153388 | 3.611488 | 0.00063 | 0.012393 | 12 months |
| Vibrio phage ICP2 | 1058.616 | 0.558087 | 0.19367 | 2.881646 | 0.005509 | 0.043967 | 12 months |
| Synechococcus phage ACG-2014c | 5727.68 | 0.562466 | 0.139305 | 4.037675 | 0.000158 | 0.007521 | 12 months |
| Pseudomonas virus PaMx25 | 5947.397 | 0.602587 | 0.166887 | 3.610757 | 0.000631 | 0.012393 | 12 months |
| Salmonella virus SE1 | 4543.887 | 0.619192 | 0.193329 | 3.202798 | 0.002195 | 0.02579 | 12 months |
| Staphylococcus virus G15 | 1918.78 | 0.634255 | 0.189427 | 3.348289 | 0.001421 | 0.019385 | 12 months |
| Escherichia virus JS09 | 27820.05 | 0.635712 | 0.204191 | 3.113321 | 0.002852 | 0.029429 | 12 months |
| Mycobacterium virus Alice | 2475.296 | 0.645548 | 0.163965 | 3.937095 | 0.00022 | 0.007747 | 12 months |
| Pseudomonas virus EL | 6351.294 | 0.685465 | 0.197934 | 3.463104 | 0.001 | 0.016224 | 12 months |
| Escherichia virus Min27 | 3923.024 | 0.70292 | 0.201846 | 3.482457 | 0.000942 | 0.015944 | 12 months |
| Escherichia virus 4MG | 3382.563 | 0.719155 | 0.220411 | 3.262783 | 0.001837 | 0.022854 | 12 months |
| Alteromonas phage vB_AmaP_AD45-P | 1790.203 | 0.762677 | 0.217152 | 3.512175 | 0.000859 | 0.015805 | 12 months |
| Mycobacterium virus Gaia | 11344.53 | 0.764673 | 0.175458 | 4.358152 | 5.32E-05 | 0.004501 | 12 months |
| Synechococcus phage S-RIM8 | 3326.444 | 0.780667 | 0.194651 | 4.010608 | 0.000173 | 0.007521 | 12 months |
| Erwinia virus Ea9-2 | 2849.026 | 0.789215 | 0.202738 | 3.892786 | 0.000255 | 0.007747 | 12 months |
| Mycobacterium virus Giles | 2471.728 | 0.799382 | 0.267998 | 2.982786 | 0.004148 | 0.036557 | 12 months |
| Helicobacter virus KHP40 | 1420.597 | 0.828164 | 0.258047 | 3.209356 | 0.002153 | 0.02579 | 12 months |
| Mycobacterium virus Bignuz | 1976.422 | 0.875041 | 0.242789 | 3.60412 | 0.000645 | 0.012393 | 12 months |
| Mycobacterium virus Sirduracell | 1435.532 | 0.885144 | 0.280616 | 3.154286 | 0.002531 | 0.027455 | 12 months |
| Mycobacterium virus Charlie | 1534.348 | 0.948924 | 0.276578 | 3.43095 | 0.001104 | 0.016683 | 12 months |
| Synechococcus phage syn9 | 3073.055 | 0.958717 | 0.229278 | 4.181459 | 9.74E-05 | 0.006864 | 12 months |
| Escherichia virus RB49 | 839.4147 | 1.003633 | 0.267702 | 3.74906 | 0.000406 | 0.010332 | 12 months |
| Mycobacterium virus Bernal13 | 3295.927 | 1.052703 | 0.231622 | 4.544909 | 2.78E-05 | 0.003915 | 12 months |
| Mycobacterium virus Muddy | 1001.92 | 1.096961 | 0.378987 | 2.89446 | 0.005316 | 0.043642 | 12 months |
| Mycobacterium virus Rey | 1803.816 | 1.113775 | 0.354785 | 3.1393 | 0.002645 | 0.027967 | 12 months |
| Escherichia virus HK578 | 23.73059 | 1.13305 | 0.359193 | 3.154434 | 0.00253 | 0.027455 | 12 months |
| Escherichia virus AR1 | 285.7573 | 1.255618 | 0.319231 | 3.933256 | 0.000223 | 0.007747 | 12 months |
| Streptomyces virus phiCAM | 836.0452 | 1.300213 | 0.388002 | 3.351043 | 0.001409 | 0.019385 | 12 months |
| Mycobacterium virus Tweety | 445.4492 | 1.398172 | 0.458795 | 3.047488 | 0.00345 | 0.034742 | 12 months |
| Mycobacterium virus Shilan | 441.3278 | 1.501607 | 0.37215 | 4.034954 | 0.000159 | 0.007521 | 12 months |
| Mycobacterium virus Redrock | 475.1814 | 1.551922 | 0.490344 | 3.164967 | 0.002453 | 0.027455 | 12 months |
| Mycobacterium virus Faith1 | 375.6739 | 1.605749 | 0.434837 | 3.692761 | 0.000487 | 0.010834 | 12 months |
| Propionibacterium virus ATCC29399BC | 121.4188 | 1.62595 | 0.559108 | 2.908117 | 0.005118 | 0.043295 | 12 months |
| Mycobacterium virus Godines | 224.5218 | 1.642281 | 0.440939 | 3.724507 | 0.00044 | 0.010332 | 12 months |
| Propionibacterium virus PHL114L00 | 91.67215 | 1.696896 | 0.454199 | 3.736019 | 0.000424 | 0.010332 | 12 months |
| Propionibacterium virus PAD20 | 104.3862 | 1.706106 | 0.494257 | 3.451861 | 0.001036 | 0.016224 | 12 months |
| Propionibacterium virus PHL060L00 | 181.7921 | 1.71153 | 0.489308 | 3.497861 | 0.000898 | 0.015834 | 12 months |
| Mycobacterium virus TM4 | 365.6142 | 2.059534 | 0.514631 | 4.001963 | 0.000178 | 0.007521 | 12 months |
| Mycobacterium virus Reprobate | 71.92575 | 2.079245 | 0.552274 | 3.764883 | 0.000386 | 0.010332 | 12 months |
| Mycobacterium virus Tiger | 60.93472 | 2.16007 | 0.495046 | 4.363369 | 5.23E-05 | 0.004501 | 12 months |
| Mycobacterium virus Taj | 79.41111 | 2.213408 | 0.56886 | 3.890951 | 0.000256 | 0.007747 | 12 months |
| Propionibacterium virus ATCC29399BT | 141.9024 | 2.24089 | 0.477149 | 4.696416 | 1.63E-05 | 0.003437 | 12 months |
| Propionibacterium virus PA6 | 58.29415 | 2.495656 | 0.452195 | 5.518977 | 8.00E-07 | 0.000338 | 12 months |

**Table S5: Infants who developed atopic disease, separated by lifestyle group.**

| | No Atopic Disease | | | Atopic Disease | | |
|---|---|---|---|---|---|---|
| | **ROC** | **OOM** | **Total** | **ROC** | **OOM** | **Total** |
| **6 weeks** | 45 | 60 | 111 | 18 | 2 | 20 |
| **6 months** | 16 | 29 | 45 | 20 | 2 | 22 |
| **12 months** | 12 | 27 | 39 | 21 | 2 | 23 |

**Tables S6: Differentially abundant enteric phage species at 6 weeks of age in infants who developed atopic disease compared to those who did not using a linear mixed effects model.**

| **Species** | **Base Mean** | **$log_2$-Fold Change** | **lfcSE** | **stat** | ***p* value** | ***p* adj** | **Visit Tested** |
|---|---|---|---|---|---|---|---|
| *Bacillus virus Grass* | 5172.92 | 0.5221 | 0.12337 | 4.2320 | 0.0000 | 0.0177 | 6 weeks |
